# Strategies to Fabricate Polypeptide-Based Structures via Ring-Opening Polymerization of *N*-Carboxyanhydrides

**DOI:** 10.3390/polym9110551

**Published:** 2017-10-25

**Authors:** Carmen M. González-Henríquez, Mauricio A. Sarabia-Vallejos, Juan Rodríguez-Hernández

**Affiliations:** 1 Departamento de Química, Facultad de Ciencias Naturales, Matemáticas y del Medio Ambiente, Universidad Tecnológica Metropolitana, P.O. Box 9845, Correo 21, Santiago 7800003, Chile; carmen.gonzalez@utem.cl; 2 Departamento de Ingeniería Estructural y Geotecnia, Escuela de Ingeniería, Pontificia Universidad Católica de Chile, P.O. Box 306, Correo 22, Santiago 7820436, Chile; masarabi@uc.cl; 3 Departamento de Química y Propiedades de Polímeros, Instituto de Ciencia y Tecnología de Polímeros-Consejo Superior de Investigaciones Científicas (ICTP-CSIC), Juan de la Cierva 3, 28006 Madrid, Spain

**Keywords:** *N*-carboxyanhydrides, ring-opening polymerization branched polypeptides, cyclic polypeptides, non-linear polypeptide architectures

## Abstract

In this review, we provide a general and clear overview about the different alternatives reported to fabricate a myriad of polypeptide architectures based on the ring-opening polymerization of *N*-carbonyanhydrides (ROP NCAs). First of all, the strategies for the preparation of NCA monomers directly from natural occurring or from modified amino acids are analyzed. The synthetic alternatives to prepare non-functionalized and functionalized NCAs are presented. Protection/deprotection protocols, as well as other functionalization chemistries are discussed in this section. Later on, the mechanisms involved in the ROP NCA polymerization, as well as the strategies developed to reduce the eventually occurring side reactions are presented. Finally, a general overview of the synthetic strategies described in the literature to fabricate different polypeptide architectures is provided. This part of the review is organized depending on the complexity of the macromolecular topology prepared. Therefore, linear homopolypeptides, random and block copolypeptides are described first. The next sections include cyclic and branched polymers such as star polypeptides, polymer brushes and highly branched structures including arborescent or dendrigraft structures.

## 1. Introduction

Since the discovery of the ring-opening polymerization of *N*-carboxyanhydrides (ROP NCAs) more than one century ago [1] a huge amount of work has been carried out to fabricate polypeptides with different functionalities as well as with variable architectures. The large variety of biomedical applications including tissue engineering, gene therapy, antibiotics or drug delivery in which synthetic polypeptides have been employed, have made of them a particularly interesting class of materials [2,3].

As a result of the large volume of work carried out in the synthesis of synthetic polypeptides today a rather large chemical diversity can be obtained. This diversity results from the combination of the twenty canonical amino acids but also to a great number of functionalization strategies. More interestingly, protein capabilities to self-assemble into precise 3D highly ordered structure have been a source of inspiration for many researchers. However, as mentioned by Deming [3] the basis for the synthesis of polypeptides able to mimic the unique properties of natural peptides and proteins entails, “in principle” the capacity to control the sequence and composition of amino acid residues along the chain as well as the chain length itself. While it is true that NCAs polymerization does not allow a perfect control over the amino acid distribution, it is worth mentioning that the advances on the polymerization mechanism permit nowadays to prepare polypeptide materials with well-defined compositions and narrow chain-length distributions. 

Two main alternative strategies have been developed for the fabrication of such complex morphologies. On the one hand, important efforts have been focused to either construct relatively simple building blocks able to self-assemble into more complex structures [4,5]. These are typically random and block copolymers that by self-assembly processes can form supramolecular objects including micelles or vesicles. On the other hand, the advances in the polymerization methodologies enabling the control over the chain length, distribution together with the “living” character of the ROP allow us to fabricate more sophisticated polymeric structures. This strategy, known as pre-assembly, resort to the synthetic tools to fabricate well-defined polymeric and usually branched polypeptides [5].

Excellent previous reviews have been devoted to describe the self-assembly processes on polypeptides and the formation of assemblies in solution (micelles, vesicles, etc.), in bulk and at surfaces [6,7,8,9,10,11]. However to the best of our knowledge the last review devoted to the pre-assembly approach, published by Hadjicrhistidis et al. [12] for the fabrication of non-linear polypeptides is outdated. Other reviews reported later either cover the synthetic advances [13,14] or focus in the preparation of polypeptides with a particular topology [15,16]. 

The main objective of this review is, therefore, to provide a general overview of the alternatives for the preparation of complex polypeptide structures discussing pioneer studies but also providing recent illustrative examples of the different strategies. This review is organized in a comprehensive way starting in Section 2 with a thorough discussion about the synthetic alternatives to prepare functionalized and non-functionalized α-amino acid NCAs. Section 3 covers those aspects related to the NCA polymerization highlighting the most recent advances reported in order to improve the control over the chain length and dispersity of the polypeptides. In this section, we will consider the strategies proposed to mediate the mechanism ROP NCA as well as those involving the improvement of the polymerization by optimizing the experimental conditions. Finally, Section 4, Section 5 and Section 6 focus on the preparation of different macromolecular polypeptide architectures starting from linear (homopolypeptides, random and block copolypeptides) described in Section 4. Whereas Section 5 will centre the attention on the synthesis of cyclic polypeptides, Section 6 is devoted to the different branched polypeptide structures ranging from star polypeptides or polypeptide brushes to highly branched dendritic graft or arborescent polypeptides.

## 2. Synthesis of α-Amino Acid *N*-Carboxyanhydrides

### 2.1. Pioneer Works about the Cyclization of Aminoacids to Synthesize NCA and Currently Employed Methodologies

The first synthesis of α-amino acid *N*-carboxyanhydrides (NCAs) reported in the literature goes back to the period comprised between 1906 and 1908 when Hermann Leuchs tried to purify *N*-ethoxycarbonyl and *N*-methoxycarbonyl amino acid chlorides via distillation, also commonly referred as Leuchs’s anhydrides [17]. After this initial experiment, a systematic investigation on the synthesis was carried out by Wessely and coworkers during the 1920s [18]. From that time, α-amino acid NCAs have become a very versatile class of monomers for the preparation of polypeptides. Two major routes have been widely employed for the preparation of *N*-carboxyanhydrides, i.e., the “Leuchs” method and the “Fuchs-Farthing” method. 

**Figure 1:**
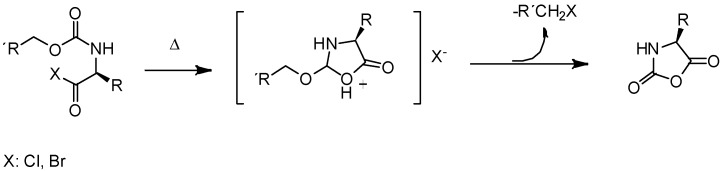
The “Leuchs method” for the preparation of α-amino acid *N*-carboxyanhydrides involves the cyclization of *N*-alkoxycarbonyl-amino acids [19].

On the one hand, pioneer works for the preparation of α-amino acid *N*-carboxyanhydrides in one single step were reported by Leuchs. Those methods involved the cyclization of *N*-alkoxycarbonyl-amino acids (Figure 1) [19]. While this reaction occurred in one single step, it required the use of high-temperature for long times, which can result in a partial decomposition of the NCAs. Interestingly, the authors found that the cyclization rate of *N*-alkoxycarbonyl amino acid halogenides depended on the carbamate substituent and increased in the following order: ethyl-methyl-allyl-benzyl [20]. As result of this, the initially employed thionyl chloride for the halogenation of the *N*-alkoxycarbonyl amino acids because of the advantage of gaseous products was replaced by phosphorus pentachloride (PCl_5_) and phosphorus trichloride (PCl_3_). The latter was more reactive and permitted the reaction to be accomplished under mild conditions. In spite of this, due to the better leaving group and better nucleophile in the dealkylation step, the most useful halogenating agent, reported later, was phosphorous tribromide, which allows the bromination process at temperatures below 25 °C [18].

In addition to the high temperatures required, a crucial aspect was also the eventual presence of impurities that accompany the synthesis of NCAs via the Leuchs method. These depended on the reagent used in the halogenation step but may include thionyl chloride, phosphorous penta- or trichloride, phosphorous tribromide, alkyl or benzylhalogenides and HCl. Bromides ions were better nucleophiles than chlorides, making phosphorous tribromide a strong halogenating agent [21]. These impurities could lead to chain termination reaction and therefore the monomers needed to be carefully purified prior to polymerization.

**Figure 2:**
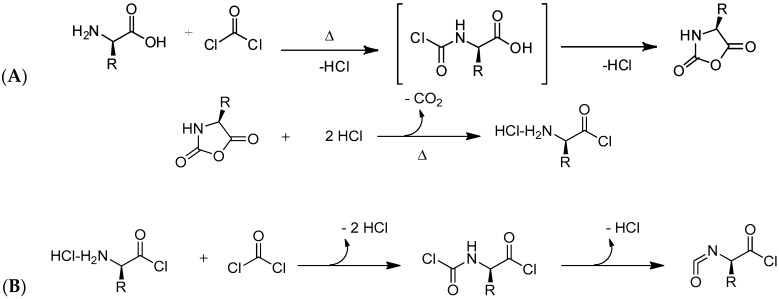
The mechanism proposed for the “Fuchs-Farthing” method [22,23,24]. (**A**) *N*-carboxyanhydrides (NCAs) formation by reaction with phosgene and (**B**) Formation of NCA and acidic decomposition.

The second synthetic approach, which is today widely employed for the preparation of NCAs, is the “Fuchs-Farthing” method [22,23,24]. This strategy allows the synthesis of pure NCA monomers with a good yield and no racemization. The mechanism proposed for the synthesis of the NCAs by this methodology is schematically shown in Figure 2A and involves the direct phosgenation of free α-amino acids (α-*N*-unprotected amino acids). Later efforts were carried out to replace the gaseous phosgene by diphosgene [25] and triphosgene [26]. These two, i.e., diphosgene and triphosgene are liquid and solid, respectively, which makes them easy to handle and allows them to be used in stoichiometric quantities. As shown in Figure 2 during the reaction HCl is produced. However, a low concentration of HCl during the NCAs synthesis is important, since HCl can lead to ring-cleavage and to the formation of unwanted α-isocyanate acid chlorides (Figure 2B). Interestingly, the solvent choice can play an important role in this NCA’s synthesis. While, in principle, low-boiling organic solvent can be used (Tetrahydrofuran, 1,4-dioxane, toluene or ethyl acetate) Kricheldorf proposed the use of 1:1 mixtures of THF or dioxane with CH_2_Cl_2_. This solvent mixture presents a reduced solubility on HCl in comparison, for example, with the pure ethers [27]. Similarly to the “Leuchs” method, several impurities can be found in the NCAs prepared via the “Fuchs-Farthing” method. These comprised, e.g., *N*-chloroformyl-amino acid chlorides and α-isocyanato-acid chlorides. The removal of these impurities generated during the synthesis was crucial since these have acidic or nucleophilic characteristics that can affect later, in the polymerization step.

The purification process for the NCA monomers involves washing NCA solutions with water or aqueous sodium bicarbonate at 0 °C in order to remove residual HCl. Subsequently, the monomer is rapidly dried [28]. While, this methodology is still widely employed, further improvements have been later reported. For instance, hydrochloride scavengers like pinene and limonene were proved effective in preventing by-product formation. This methodology reported by Smeets et al. was demonstrated to be particularly well adapted for the synthesis of l-leucine NCA (Leu-NCA) [29]. A more recent improvement in the purification process was reported recently by Deming et al. [30]. They used a rapid and general method i.e., flash column chromatography. This technique was effective at removing all common impurities from NCAs and was found to work for a variety of NCAs, including those synthesized using different routes as well as those bearing either hydrophilic or hydrophobic side chains (especially interesting for those that cannot be recrystallized).

### 2.2. Functional NCA Monomers

Many studies have been devoted to the fabrication of polypeptides using natural occurring polypeptides. Natural occurring aminoacids without functional side chain groups can be easily polymerized the fabrication of polypeptides bearing side-chain functional groups required additional considerations. However, for many different applications polypeptides with side functional groups are required. In those cases, the amino acid side-chain groups require the use of, for instance, additional protection/deprotection chemistry to be polymerized. In view to enlarge the variety of side functional group a number of different strategies have been developed during the last decade to incorporate novel functional groups either in the NCA monomers or by post-modification of pre-formed polypeptides. This section will be thus devoted to describing illustrative examples for the preparation of functional NCA monomers.

#### 2.2.1. Protected Functional Groups Using Natural Occurring Amino Acids

**Figure 3:**
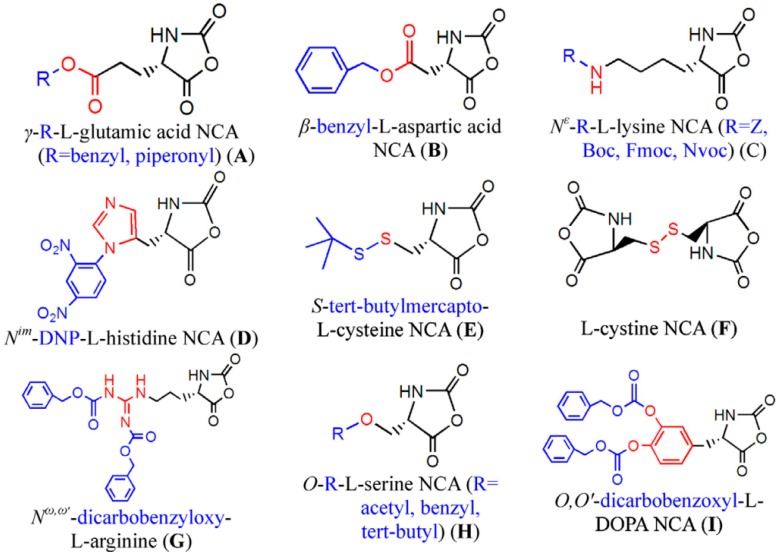
Functional NCA monomers containing a protected pendant functional group. Reproduced with permission from reference [31].

As mentioned above, the first alternative to fabricate functional polypeptides is by direct polymerization of NCA monomers that contain protected pendant groups. Protective pendant groups have been employed to block functional groups such as amine, carboxylic acid, hydroxyl, imidazole, thiol, and guanidine during the polymerization step followed by deprotection. Figure 3 shows several examples of functional NCA monomers that require protective chemistry to be polymerized. Among the depicted NCAs PGlu, PAsp, PLys are without any doubt the most extensively employed. The strategy to prepare the protected polypeptides involves, as depicted by Chao et al. [13,31] four different steps. In the case of PGlu and PAsp the β/ω carboxyl groups are first protected by forming typically a copper complex that protects the α-position. Then, the NCA is synthesized and polymerized by using any of the mechanisms (see next section) and finally the protective groups are removed. Carboxylic acid groups are usually protected using benzyl groups and the resulting polypeptide can be deprotected using different alternatives such as hydrogenation, using basic conditions or using strong acids. The same strategy can be employed for PLys but, in this case the ω-amino group is protected. Different protective chemistries have been reported for the case of Lysine and protective groups include: *tert*-butoxycarbonyl (Boc), benzyloxycarbonyl (Z), trifluoroacetyl (TFA), 6-nitroveratryloxycarbonyl (Nvoc) and 9-fluorenylmethoxycarbonyl (Fmoc) [32].

#### 2.2.2. Other Side Chain Functionalized NCAs

**Figure 4:**
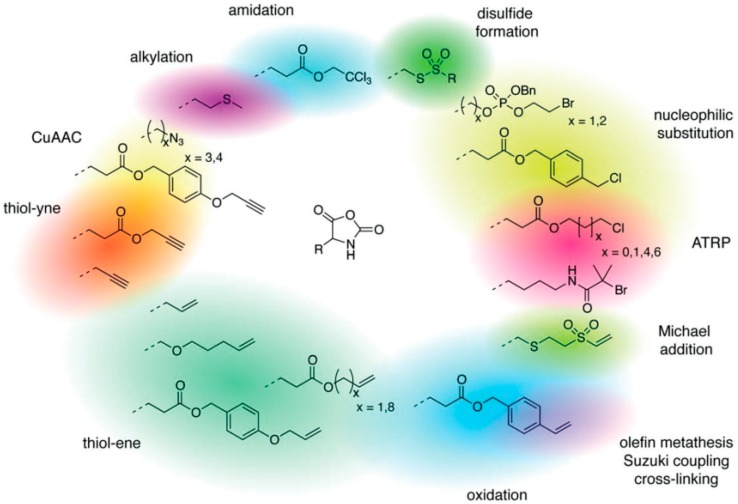
General overview of the functional NCAs and the corresponding chemical modifications that they may undergo. Reproduced with permission from reference [14].

In order to fabricate polypeptides with additional functionalities, novel NCA monomers have been prepared. As illustrated in Figure 4, a wide variety of side chain functional groups of NCAs leads to a wide variety of monomers that can be summarized in the following general groups: NCAs for cycloaddition reactions, alkene NCAs, halogenide NCAs, reactive ester NCAs, S-sulfonilester NCAs [13,14]. Selected examples with the precise chemical structure and the corresponding reference are equally provided in Table 1.

NCAs have been extensively functionalized, for instance, with side-chain functional groups that can undergo alkyne-azide Huisgen cycloadditions. Since the pioneer work of Hammond et al. [33,34,35,36] incorporating propargyl groups, other reports have introduced precursors for the same type of reaction in order to carry out pegylation or glycosylation reactions [37,38]. Also NCA monomers with chloro- side groups [39] as well as azide groups [40] have been prepared for their post-modification via a Huisgen cycloaddition. 

Another class of reactions that can be carried out on functionalized NCAs is the thiol-ene reactions. These NCAs are designed to bear a double bond as side-group [41,42,43,44]. An interesting class of functional NCAs is represented by the incorporation of saccharides, and in particular glucose thus leading to glycosylated peptides. Interestingly, these NCAs are polymerized and do not require any additional modification.

Finally, pH response provided by the carboxylic acid groups (e.g., in the case of l-glutamic acid) or the amine groups (e.g., in the case of l-Lysine) as well as thermal and photoresponsive polypeptides have been fabricated by introducing the appropriate functional groups. For example, Chen et al. [48] and Fu et al. [49] prepared functionalized NCA bearing oligoethylene glycol functional groups known for changing the solubility by increasing the temperature. Photoresponsive polypeptides were synthesized by Liu et al. [50] and Yin et al. [51]. For instance, Liu et al. [50] reported the synthesis of a photoresponsive S-(o-nitrobenzyl)-l-cysteine *N*-carboxyanhydride (NBC-NCA) monomer employed for the synthesis of poly(*S*-(*o*-nitrobenzyl)-l-cysteine)-*b*-poly(ethylene glycol) (PNBC-*b*-PEO) block copolymers. The β-sheet conformational PNBC block presented a thermotropic liquid crystal phase behavior, and the crystallinity of the PEO block was progressively suppressed over the PNBC composition. Moreover, the characteristic absorption peaks of these copolymers at about 310 and 350 nm increased over UV irradiation time indicating that the *o*-nitrobenzyl groups from the copolymers were gradually photocleaved until the completion of photocleavage. The PNBC-*b*-PEO copolymers prepared self-assembled into spherical nanoparticles in aqueous solution, presenting a photoresponsive self-assembly behavior, together with a size reduction of nanoparticles after irradiation. 

**Figure 5:**
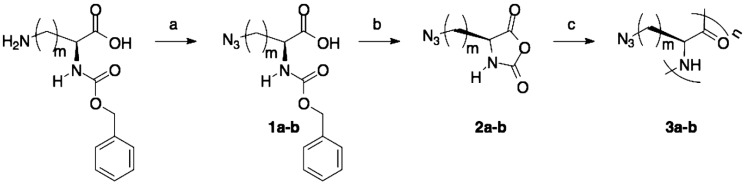
Synthesis of azide-containing NCAs. (a) Imidazole-1-sulfonyl-azide·HCl, CuSO_4_·5H_2_O, K_2_CO_3_, 1:1 THF:H_2_O, 16 h (88% yield, 1b). (b) Ghosez’s reagent, THF, 21 °C, 48 h (67% yield, 2b). (c) (PMe_3_)_4_Co, THF, 21 °C, 1 h (96% yield, 3b). 2a = l-azidonorvaline-*N*-carboxyanhydride (m = 3, Anv NCA), 2b = l-azidonorleucine-*N*-carboxyanhydride (m = 4, Anl NCA), 3a = poly(l-azidonorvaline), poly(Anv), 3b = poly(l-azidonorleucine), poly(Anl). Reproduced with permission from reference [40].

Rhodes et al. [40] prepared azide-containing NCAs, using l-lysine and l-ornithine as starting materials since their side-chain amine groups can be readily converted to azides in a single step. As depicted in Figure 5, azido amino acids were prepared from the *N*^α^-carboxybenzyl (Cbz) protected amino acids using previously reported procedures. These derivatives were then directly converted to NCAs, via the acid chloride using the Ghosez’s reagent, maintaining the integrity of the azide functionality. Polymerizations of Anl-NCA and Anv-NCA using (PMe_3_)_4_Co in THF proceeded readily at ambient temperature to give the corresponding homopolypeptides, poly(Anl) and poly(Anv), with complete monomer conversions and no reactions at the side-chain azido groups.

**Figure 6:**
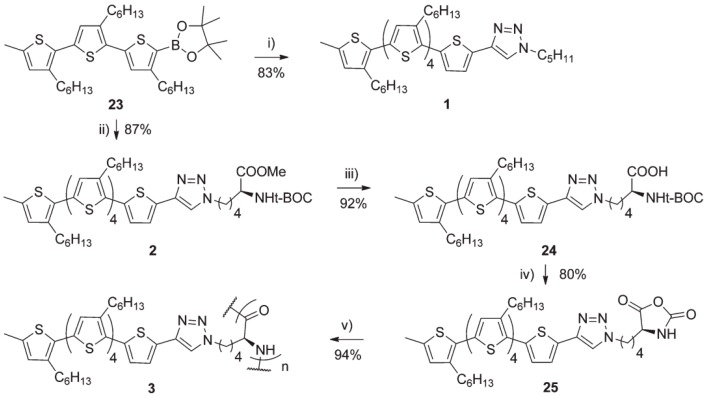
Synthetic route for the preparation of Semiconductor Functionalized Peptide. Reagents and conditions: (i) 12, Pd(PPh_3_)_4_, K_2_CO_3_, DME, H_2_O, 130 °C, 1 h; (ii) 15, Pd(PPh_3_)_4_, Cs_2_CO_3_, toluene, reflux, 16 h; (iii) LiOH, THF, H_2_O; (iv) Et_3_N, triphosgene, EtOAc; (v) HMDS, THF, 32 h. Reproduced with permission from reference [53].

Finally, another interesting example for the fabrication of conductive polypeptides was reported by Holmes et al. [53]. As shown in Figure 6, they synthesized a hexithiophene functionalized Lys-NCA and polymerized it. The materials obtained presented hierarchical self-assembled structures that finally resulted in interesting properties organic photovoltaic applications as well as the preparation of organic field transistor devices.

## 3. Polymerization of α-Amino Acid *N*-Carboxyanhydrides

### 3.1. General Mechanisms and Historical View

**Figure 7:**
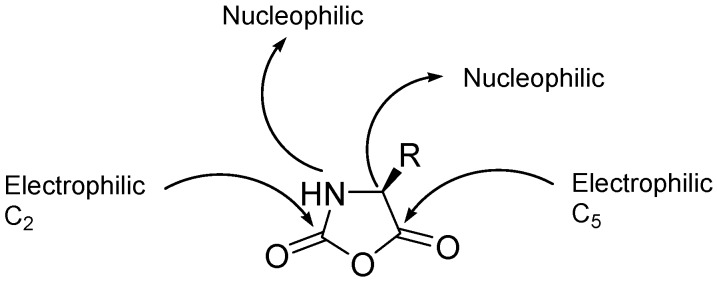
Structure and reactive centers in α-amino acid *N*-carboxyanhydrides.

The chemical structure of α-Amino acid *N*-carboxyanhydrides (NCAs) is shown in Figure 7. NCAs are characterized by four reactive sites, two nucleophilic sites (after deprotonation of the NH and CH groups) and two electrophilic groups (C-2, C-5). As a result, the ring of the NCAs can be opened and NCAs can be polymerized via several concurrent mechanisms. Therefore, the preparation of well-defined polypeptides in terms of chain-length and dispersity is not an easy task.

**Figure 8:**
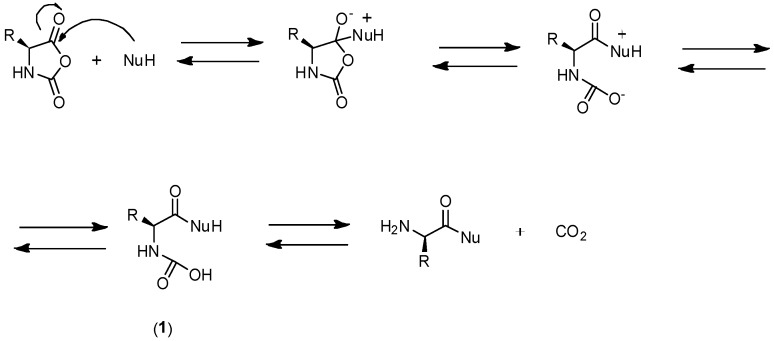
Polymerization via the “amine mechanism” (also called the “protic mechanism”) reported by Wessely and by Watson et al. [57].

**Figure 9:**
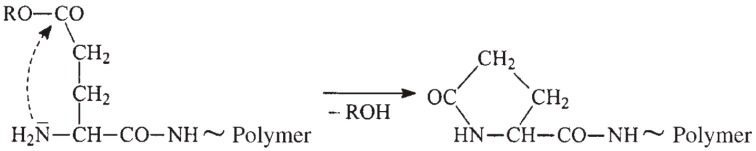
Intramolecular termination step that is typical for poly(γ-*O*-allyl-l-glutamate)s. Reproduced with permission from reference [58].

There exist two main mechanisms generally established for NCA’s polymerization, i.e., the “amine mechanism” and the “activated monomer mechanism”. It is outside of the scope of this review to thoroughly analyze the polymerization mechanism that can be found in excellent reviews reporting the synthesis and the polymerization of NCAs have been written by Kricheldorf, Bamford, Hadjichristidis, and Deming among others [12,18,26,54,55,56]. Nevertheless, a brief explanation will be provided. On the one hand, the polymerization via the “amine mechanism” (which is also known under the name “protic mechanism”) is initiated by protic nucleophiles such as primary amines and was first reported by Wessely and by Watson et al. [57]. In this mechanism, shown in Figure 8, the primary amine reacts with the C-5 in the NCA monomer. The NCA’s ring opens, carbon dioxide is removed and a molecule with a new primary end group is formed for further reaction with other NCA. As will be explained later, the amine initiator used in this case is incorporated in the growing chain in contrast to the initiators employed in the “activated monomer mechanism” that served to extract the proton of the activated NCAs. It is worth mentioning that primary amines, such as e.g., *n*-butylamine and *n*-hexylamine, are highly nucleophilic compared to the reactive amine chain ends. This is a priori an interesting aspect since the initiation process is much faster than propagation. This is a requisite for a control of the molecular weight and to obtain narrow polydisperse polymers. However, some other termination reactions present may limit the fabrication of high molecular polypeptides. For example, cyclization of chain ends typically observed in the polymerization of poly(l-glutamates) enable only the formation of polypeptides with molecular weights in the range of degree of polymerization (DP) < 150–200 (Figure 9) [58].

**Figure 10:**
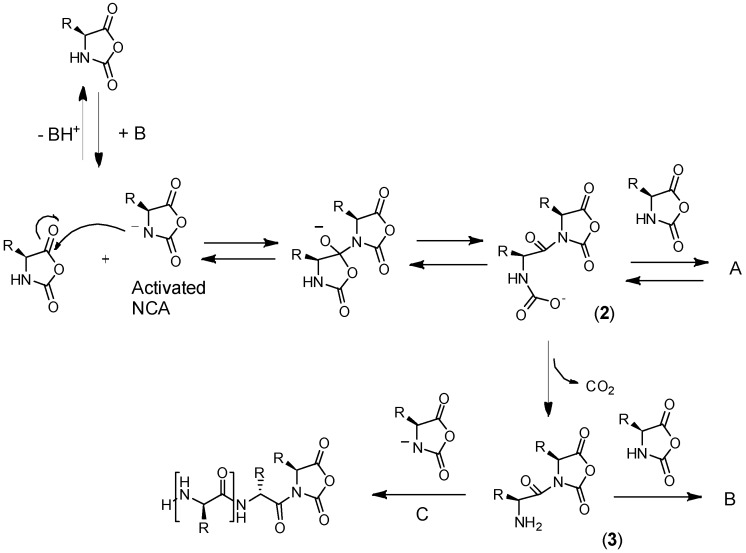
The polymerization of NCAs under the action of aprotic bases is thought to proceed via so-called “activated-monomer mechanism” [22,23].

**Figure 11:**
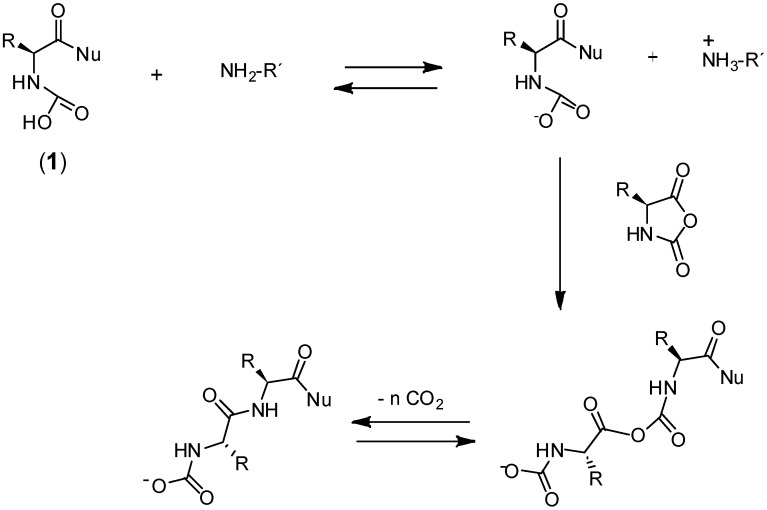
Scheme of the side reaction that usually accompanies the polymerization of NCAs initiated by primary amines commonly called the “carbamate mechanism”.

The polymerization of NCAs typically occurs through the “activated-monomer mechanism” when aprotic bases are employed (Figure 10) [59,60]. The use of a strong base deprotonates the NCA, which becomes nucleophilic and, under these conditions, is able to react with another NCA. Three different paths followed by the activated NCA monomer. In the first situation, the dimer obtained can, upon, ring-opening, protonation, and elimination of CO_2_ led to an amino-terminal group that follows from now the amine mechanism (Route B). The second situation involves the attack by the activated monomer of the electrophilic *N*-acyl-NCA end group (Route C). Finally, the nucleophilic carbamate group (Intermediate 2) can react according to a side reaction, i.e., the carbamate mechanism shown in Figure 11 (also Route A in Figure 10) by an attack of another NCA monomer. 

As a result, initiators that polymerize NCAs according to the activated monomer mechanism are typically tertiary amines such as triethylamine or alkoxide anions. The use of secondary amines requires further considerations since these initiators can react with the NCAs in two different ways. On one hand, secondary amines can act as nucleophiles and therefore, with the C_5_ attack of the NCA a thereon follow the “amine mechanism”. On the other hand, however, they may act as a base, attacking the *N*-proton and following the “activated-NCA” mechanism. In conclusion, when using secondary amines, the initiation mode will depend on the nucleophilicity/basicity (Nu/B^−^) ratio, i.e., secondary amines with low Nu/B^−^ ratio, such as cyclohexylamine or di-*n*-propylamine, polymerize NCAs according to the activated monomer mechanism and secondary amines with a high Nu/B^−^ ratio behave as primary amines.

Pioneer works carried out by Blout and Karlson evidenced for the first time that initiation of the polymerization of γ-benzyl-l-glutamate *N*-carboxyanhydride resulted in very high molecular weight polypeptides [61]. The initiators used in this research were aprotic bases, which represent the second possibility for initiate the ring opening NCA polymerization. 

As has been mentioned above, there exists a side reaction which accompanies the polymerization of NCAs initiated by primary amines known as “carbamate mechanism” (Figure 11). This mechanism takes place when the primary amine is basic enough to deprotonate the intermediate carbamic acid (Intermediate 1). The molecule generated can react via a nucleophilic reaction with an NCA monomer resulting in an intermediate anhydride. A new peptide bond is then formed after decarboxylation and, subsequently, the polymerization can proceed. Whereas in the case of NCAs’ polymerization with primary amines, the “carbamate mechanism” only plays a minor role, this side reaction can be an important limitation in the “activated monomer mechanism”. 

A huge amount of work in the fabrication of polypeptides, copolypeptides and even some branched structures by any of the mechanisms proposed above has been reported by Sela and Katchalski [62,63]. Their unique contributions, that started back to 1950–1960, settle the basis for the subsequent developments in NCA ROP.

### 3.2. Improvements in the Polymerization of α-Amino Acid N-Carboxyanhydrides

The major limitation of both the standard amine initiated and the activated monomer mechanism for the NCA polymerization is the “carbamate mechanism”. In order to improve this drawback further research focused on the design of novel polymerization alternatives attempting to reduce, or even completely avoid, these side reactions. The different alternatives proposed to overcome this issue are depicted in this section. 

#### 3.2.1. Strategies to Improve Control over Chain Length and Dispersity: Role of the Initiator and Mediation of the Ring-Opening Polymerization 

**Figure 12:**
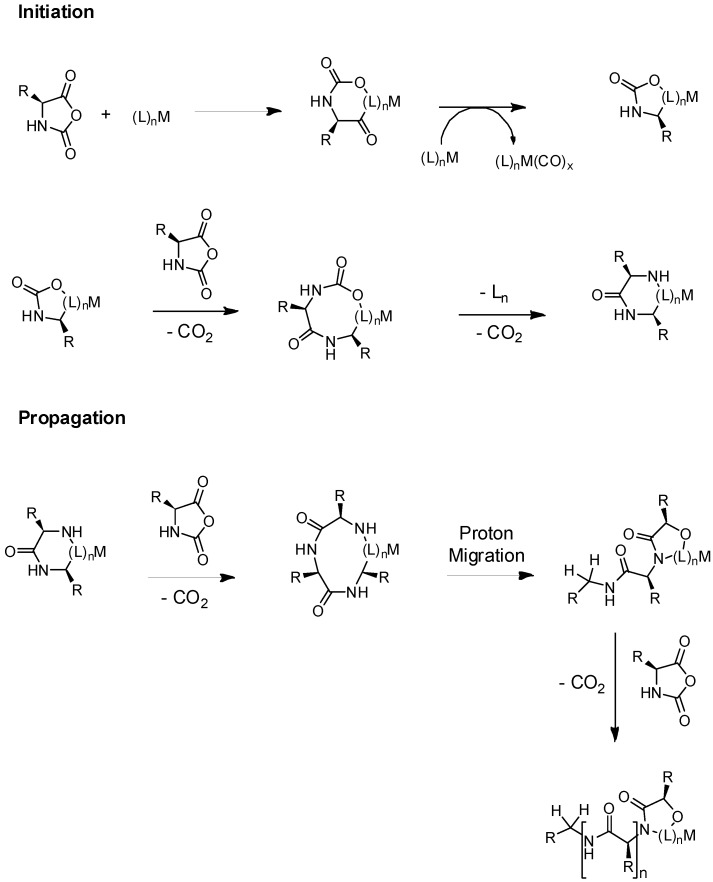
Propagation Reactions of the Mechanism for the Polymerization of NCAs with bipyNi (COD) (bipy) 2,2-bipyridyl, (COD 1,5-cyclooctadiene) or (PMe_3_)_4_Co complexes proposed by Deming et al. [64,65,66].

One of the pioneer works reported in order to improve the polymerization mechanism was reported by Deming et al. [64,65,66]. Their approach overcomes several drawbacks of the traditional NCA initiator systems, both in terms of chain length control and dispersity. In their strategy, they substituted the amines typically employed in the initiation of the ROP NCAs by transition-metal complexes as active species to control the addition of NCA monomers to the polymer chain-end. As a result, the NCA polymerization with these transition metal complexes follows an alternative mechanism permitting the synthesis of polypeptides with predictable molecular weights and narrow polydispersities. The proposed mechanism is outlined in Figure 12.

As will be thoroughly described in the next sections, the control over the polymerization process is a crucial requirement to fabricate more complex structures. For instance, Deming et al. synthesize homo- and block-copolypeptides with predictable molecular characteristics and low dispesities (DIs) based on the use of zero-valent nickel complex bipyNi (COD) (bipy) 2,2-bipyridyl, (COD 1,5-cyclooctadiene). Later, they reported the use of cobalt initiators of the (PMe_3_)_4_Co type that resulted equally efficient [67]. Although this strategy presented significant advantages, one of the major drawbacks was that the metal ions employed need be conveniently removed from the polymers. The authors reported that simple precipitation or dialysis of the sample after polymerization was enough to readily remove the metal ions.

**Figure 13:**
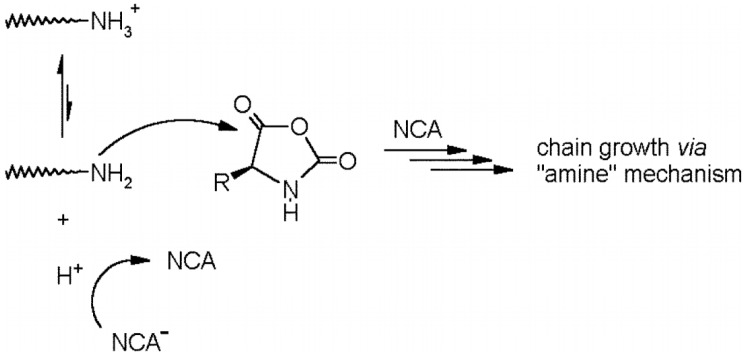
Tentative mechanism of the ring-opening polymerization of NCAs (ZLLys, R = (CH_2_)_4_NHC(O)OCH_2_C_6_H_5_) using primary amine–hydrochlorides (chloride ions omitted). Reproduced with permission from reference [68].

A second strategy was reported by Schlaad’s group [68]. They proposed to control the protonation of the amine initiator group to form hydrochloric salts as a strategy to reduce secondary reactions. As shown in Figure 13, the acidic conditions in the system produced the elimination of CO_2_ from the reactive intermediate and more importantly, suppressed the formation of unwanted NCA anions. Consequently, as soon as free amine reacted with NCA, the resulting amine end-group on the product was immediately protonated and prevented further reaction. According to these findings, the dissociation of the hydrochloride released the propagating primary amine and a proton, which avoided chain growth via the “activated monomer” mechanism. In agreement with the studies carried out by Knobler et al. [69,70] only one NCA molecule reacted with such salts, without propagation, since the hydrochloric salt of the primary amine formed was less nucleophilic than the parent amine, which effectively halted the reaction after a single NCA insertion by the formation of an inert amine hydrochloride in the product. The dormant amine hydrochloride species was favored in this equilibrium, and therefore, the free amines were reactive for only a very short time and could not propagate. The authors demonstrated the controlled polymerization of ZLLys-NCA in *N*,*N*-dimethylformamide (DMF) 40–80 °C using PS_52_–NH_2_·HCl as a macroinitiator. As a conclusion, although these polymerizations were slow compared to the amine-initiated polymerization, the resulting PS-*b*-PZLLys block copolymers exhibited a very narrow molecular weight distribution, close to a Poisson distribution (PDI < 1.03). These distributions were much narrower than those obtained using the free amine macroinitiator, which argues for diminished side reaction on the polypeptides synthesis.

More recently, Conejos-Sánchez et al. [71] resorted to the use of ammonium salts with non-nucleophilic tetrafluoroborate anions as initiators for the ring opening polymerization of α-*N*-carboxyanhydrides (NCAs). This methodology permitted the synthesis of polyglutamates with defined molecular weights (up to 800 units), low dispersities (<1.2), controlled chain end functionality, an adequate stereoselectivity and absence of any trace of toxic impurity thus allowing the use of the polypeptide synthesized for their use in biomedical applications. 

**Figure 14:**
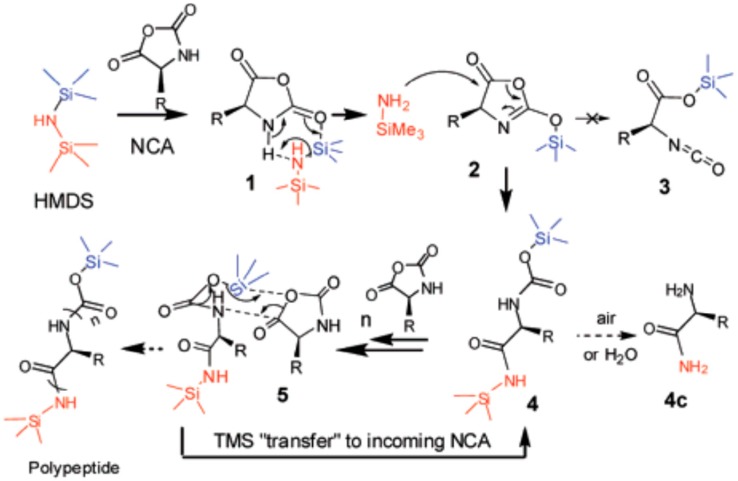
Hexamethyldisilazane (HMDS)-mediated NCA polymerization through trimethylsilyl (TMS) carbamate group. Reproduced with permission from reference [72,73].

The second alternative to control the macromolecular characteristics of the synthesized polypeptides is based on the mediation of the mechanism of the NCA ROP. Using this alternative, Lu et al. [72] found that NCA polymerizations mediated by hexamethyldisilazane (HMDS) remained controlled and living. The mechanism proposed by the authors is shown in Figure 14. The authors evidenced that the initiation step involved the cleavage of the N−Si bond of HMDS and the formation of a trimethylsilyl carbamate (TMS-CBM) terminal group. Therefore, the polypeptide chains were propagated through the migration of TMS of the TMS-CBM end group to the incoming monomer and formed a new TMS-CBM terminal group. As a result, this organosilicon reagent mediated NCA polymerization offered a metal-free strategy for the convenient synthesis of homo- or block polypeptides with predictable molecular weights and narrow molecular weight distributions.

**Figure 15:**
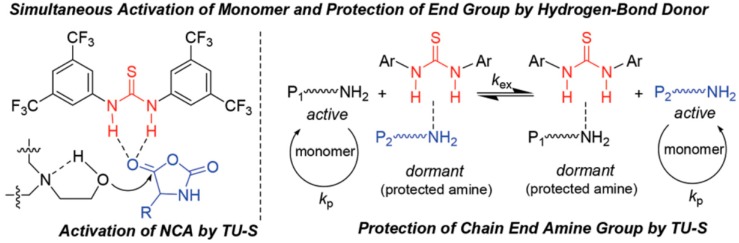
Strategy reported by Zhao et al. for the living polymerization of α-amino acid NCAs. Reproduced with permission from reference [74].

A similar strategy was recently employed by Zhao et al. [74]. They reported, based on hydrogen-bonding organocatalysis, the living ring-opening polymerization of *N*-carboxyanhydride of α-amino acids using aminoalcohols as initiators in the presence of *N*,*N*′-bis[3,5-bis(trifluoromethyl)phenyl]thiourea (TU-S). As it is shown in Figure 15, the thiourea provided, through hydrogen bonding, simultaneous activation of NCA monomers/reversible deactivation of polymer chain-ends/silencing of the tertiary amine and thus allowed the polymerization to proceed in a highly controllable mode. For example, by using *N*,*N*-dimethyl ethanolamine (DMEA), as an initiator in the presence of TU-S, a series of well-defined linear polypeptides with differently designed M_ns_ (3.01 × 10^4^–18.10 × 10^4^) and low PDI values (1.02–1.05) were successfully synthesized. 

Finally, a recent development described in the literature concerning the NCA mediation was described by Yuan et al. [75] They reported the ring-opening polymerization (ROP) of α-amino acid *N*-carboxyanhydrides (NCAs) mediated by trimethylsilyl sulfide (S-TMS). According to the authors, phenyl trimethylsilyl sulfide (PhS-TMS), an inexpensive and commercially available compound, mediates rapid ROP of a broad scope of NCA monomers, producing functional poly(amino acids) (PAAs) with controllable molecular weights (MWs), narrow dispersity index (DI), and an in-situ generated phenyl thioester group at the C-terminus (PAA-SPhs). PhS-TMS, due to their rapid chain initiation, ensures a living polymerization with improved control. Their mechanistic studies suggested that the reactive trimethylsilyl carbamate (TMSC) was generated during the chain initiation and continued to regulate the chain propagation through a TMS transfer process. 

#### 3.2.2. Optimization of the Experimental Conditions for the NCA Polymerization 

Hadjichristidis and co-workers [76] assumed that most of the problems found in the traditional NCA polymerization were related to impurities traces present in the reaction media. Their strategy involved the polymerization of NCAs with primary amines, e.g., *n*-hexylamine and 1,6-diaminohexane, strong nucleophiles, which are known to direct the reaction through the “normal-amine route” together with high vacuum techniques (HVT) in order to create and maintain the necessary conditions for the NCAs’ living polymerization. The polypeptides produced by this method could be prepared with controlled chain lengths, producing narrow chain length distribution. HVT ensured that all reagents, and also the reaction environment, are completely impurity-free in all steps of the synthesis. For this purpose, they fabricated reactors equipped with break-seals along with magnets covered with glass, and constrictions, for the reagents addition and removal of intermediate products. Controlled polymerization of NCAs under high vacuum was later confirmed by Messman and co-workers [77].

A decrease in the reaction temperature can be also employed to control the NCA polymerization. Vayaboury et al. [78] studied the *n*-hexylamine-initiated polymerization of *N*^ε^-trifluoroacetyl-l-lysine *N*-carboxyanhydride in *N*,*N*-dimethyformamide by nonaqueous capillary electrophoresis (NACE). A polypeptide with a broad molecular weight distribution was obtained and side reactions were clearly identified for polymerizations carried out, either at room or higher temperatures (Table 2). However, when the polymerizations were carried out at 0 °C almost no dead polymer was formed. The temperature effect is not unusual, similar trends were found in cationic and anionic vinyl polymerizations. At elevated temperatures, the side reactions have activation barriers similar to chain propagation. When the temperature of the reaction is lower, it appears that the activation barrier for chain propagation becomes lower than the necessary for side reactions and, eventually, chain propagation kinetically dominates the reaction.

More recently Habraken et al. [79,80] examined how experimental factors including the pressure and temperature affect the polymerization of various NCA monomers, i.e., γ-benzyl-l-glutamate (BLG), *N*^ε^-benzyloxycarbonyl-l-lysine (ZLL), l-alanine (Ala), β-benzyl-l-aspartate (BLA), *O*-benzyl-l-serine (BLS), and *O*-benzyl-l-threonine (BLT). They found that the studied NCAs could be divided into two groups: in the first group, monomers of BLG, ZLL and Ala polymerized considerably faster when a lower pressure of 1 × 10^5^ bar was applied. Matrix-assisted laser desorption/ionization time of flight mass spectrometry (MALDI-ToF-MS) analysis confirmed that the formation of side products for these monomers mostly started after full monomer conversion. The second group of monomers, i.e., BLA, BLS and BLT, polymerized considerably slower than the first group and no effect was observed from the lower pressure. On the other hand, the number of side reactions was significant at 20 °C, so that the polymerizations for the latter monomers should preferably be done at 0 °C. Their results indicated that the γ-benzylester cleavage by terminal amine (backbiting) is the major contamination for Bn-Glu-NCA polymerization and in the case of Bn-Asp NCA, a more intricate scenario was revealed at elevated temperatures, which showed impurities that comprise side-chain ester cleavage and formamide end-capping from DMF. 

A novel interesting strategy to fabricate polypeptides was reported by Heise et al. [81]. They reported the first example of UV-initiated synthesis of polypeptides from NCAs. The active initiator cyclohexylamine was produced in-situ by the UV-induced breakdown of photoamine generators. The authors carried out real-time FTIR and MALDI-ToF-MS analyses in order to obtain evidence for the proposed photoinitiation mechanism as well as the attachment of the active initiator to the polypeptide chain. 

#### 3.2.3. Alternative Cyclic Monomers to NCAs for the Fabrication of Polypeptides

**Figure 16:**
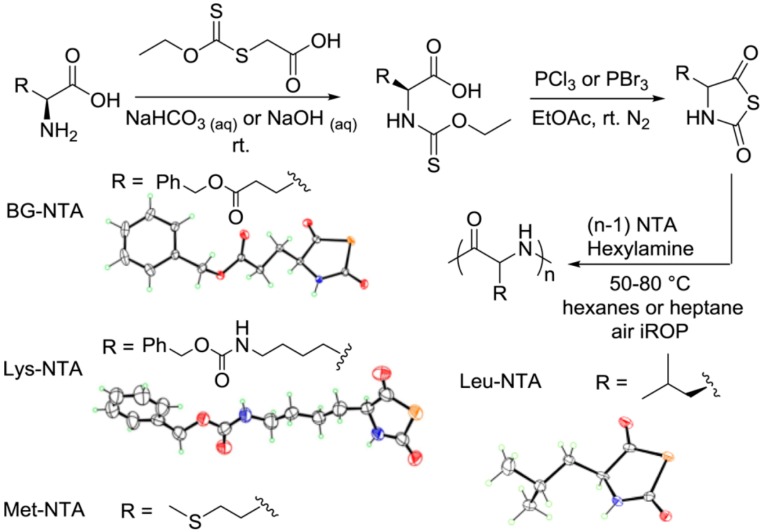
Synthetic Route towards *N*-thiocarboxyanhydrides (NTAs) according to Cao et al. [82].

Cao et al. [82] demonstrated that interfacial ring-opening polymerizations (iROP) of α-amino-acid derived *N*-thiocarboxyanhydrides (NTAs) in hexane or heptane suspension, using soluble primary amine initiators, can be employed to synthesize polypeptides with controlled molecular weight and low-to moderate molecular weight distribution under mild conditions. According to the authors, the NTA monomers improved both moisture and thermal stability in comparison to *N*-carboxyanhydrides (NCAs). These unique characteristics result in long shelf life and permit the polymerization to occur quantitatively in open air. A scheme of the synthetic strategy to prepare NTA’s monomers is depicted in Figure 16.

#### 3.2.4. Improvement of the Polymerization Kinetics While Maintaining the Living Features

A reduction of the polymerization kinetics has a positive influence on the control of the polymerization. However, the synthesis of longer polypeptides can be a rather long process. In order to accelerate the kinetics while maintaining the living feature, Zou et al. [83] reported a straightforward method to enhance the polymerization rate while maintaining the living features of the polymerization by simply using N_2_ flow during the NCA ROP. 

Although the influence of CO_2_ in NCA ROP was studied 50 years ago when it was observed that the immediate removal of CO_2_ affected the kinetics of polymerization [84], the “livingness” of the NCA ROP with removal of CO_2_ from the reaction was not demonstrated. Zou et al. confirmed the advantages of N_2_ flow methods and confirmed the living characteristics for NCA ROPs by employing γ-benzyl-l-glutamate (BLG-NCA) as a well-studied monomer and *n*-hexylamine as initiator. Compared to the methods for the preparation of polypeptides from NCAs using primary amines as initiators without catalyst activation, the use of N_2_ gas presented several important advantages: (1) promoted polymerization rates, to allow NCA conversions to reach >95% in a matter of hours rather than the multiple day time period that is required typically; (2) glovebox-free operation in a normal fume hood, to increase the convenience, decrease the time, and allow for greater variation of the reaction conditions, for instance the temperature; (3) control over the polymerization rate by altering the flow rate of nitrogen; (4) maintenance of the living features of NCA ROP even at high conversions and high monomer:initiator feed ratios [83].

**Figure 17:**
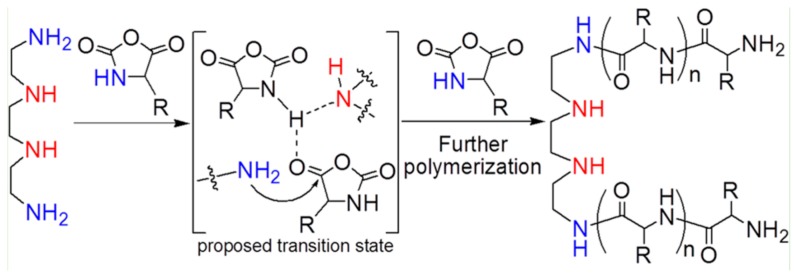
Accelerated amine mechanism trhough monomer activation (AAMMA). Reproduced with permission from reference [86].

More recently, an interesting fast and living ROP NCA mechanism was reported by Zhao et al. [85] Their methodology is based on an “alliance” of primary and secondary amine initiators and allows for fast and living ring-opening polymerization (ROP) of α-amino acid *N*-carboxyanhydrides (NCAs) at room temperature. The mechanism proposed, illustrated in Figure 17, indicates that an initiator like triethylaminetriamine (TREN) both its core tertiary amine and the three growing primary amines act cooperatively and in synergy to bring about a fast and yet “living” polymerization of NCAs through the so-called accelerated amine mechanism through monomer activation (AAMMA). They later show that primary and secondary amines can also act cooperatively and in synergy to trigger the “living” polymerization of NCAs [86]. In contrast to conventional amine-mediated NCA polymerizations, these “allied” amines did not require low reaction temperatures to prevent side reactions from occurring, and they afforded well-defined polypeptides at room temperature. This unexpected finding and its associated AAMMA mechanism provided a new strategy to develop effective metal-free initiators for NCA polymerization.

## 4. Linear Side Chain-Functionalized Polypeptides 

### 4.1. Homo and Copolypeptides Bearing Side-Chain Functional Groups

#### 4.1.1. Chemical Modification of Homopolypeptides

**Figure 18:**
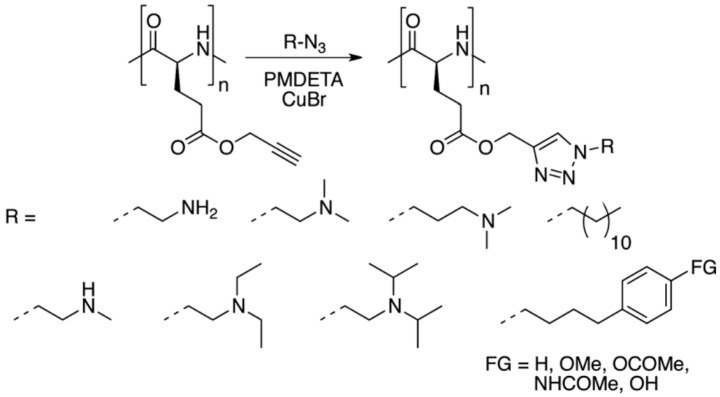
Poly(γ-propargyl-l-glutamate) clicked to various amines to obtain polycationic [87] or hydrophobic polymers [88].

Two main strategies can be followed in order to modify polypeptides to render them functional. On the one hand, functional homopolypeptides can be prepared using NCAs bearing side functional groups. On the other hand, by chemical post-modification of the preformed polypeptides [31]. While it is true that both methodologies have been reported, the second alternative is by far the most extended approach. A large variety of examples have been reported in which NCAs can be designed to incorporate different functional groups including halogen-, alkyne, azide-, allyl- (for Huisgen cyclization reactions), vinyl benzyl-groups (for thiol-ene reactions) that can undergo further modification but also to introduce initiators for additional polymerization steps [33,34,35,36,52]. One of pioneer works of post-modification were reported by Hammond et al. [33,87,88]. They employed click chemistry to prepare poly(γ-propargyl-l-glutamate) that permits to chemically modify the alkyne side chain groups with a polyethylene glycol azide (PEG-azide) to render the polypeptide hydrophilic (Figure 18). 

**Figure 19:**
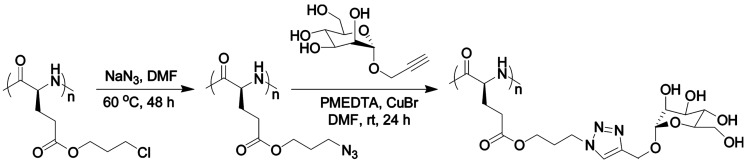
Post-modification of the side-functional groups to introduce Mannose. Reproduced with permission from reference [39].

Another interesting example was reported by Zhang et al. [39]. They described the synthesis of a series of poly(γ-chloropropyl-l-glutamates) (PCPLG) with controlled polymer molecular weight and molecular weight distribution by hexamethyldisilazane (HMDS)-mediated ring-opening polymerization (ROP) of γ-chloropropyl-l-glutamic acid based *N*-carboxylanhydride (CP-NCA). Interestingly, conformational analysis of these polypeptides revealed that the polymers adopted α-helical conformations both in solution and the solid state. As a result, their helical surfaces could be readily decorated. The authors demonstrated a quantitative derivatization of the PCPLG side chains with azido functional groups (Figure 19). Subsequent side-chain conjugation with mannose moieties via copper-mediated [2 + 3] alkyne-azide 1,3-dipolar cycloaddition afforded water-soluble mannose-polypeptide conjugates with quantitative grafting efficiency occurring under mild conditions. 

Xiao et al. [35] presented a straightforward route for the preparation of glycopolypeptides with highly effective “glycosylation” by click postpolymerization modification (grafting efficiency 60–100% depending on the feed ratio). For that purpose, they synthesized an alkyne-substituted *N*-carboxyanhydride (NCA) monomer that was, in turn, polymerized to afford the polypeptide with “clickable” alkyne pendants. The alkyne-functionalized polypeptide was then “glycosylated” by click reaction of different sugar azides to the alkyne pendant groups with high efficiency. Later, the same group [9] reported the synthesis of amphiphilic homoglycopolypeptides by a combination of NCA polymerization and click chemistry to yield a well-defined polypeptide having an amphiphilic carbohydrate on its side chain. The amphiphilicity of the carbohydrate was achieved by incorporation of an alkyl chain at the C-6 position of the carbohydrate, thus, also rendering the homoglycopolypeptide amphiphilic. The homoglycopolypeptide formed multimicellar aggregates in water above a critical concentration of 0.9 μM due to phase separation. According to the authors hydrophobic interactions of the aliphatic chains at the 6-position of the sugar moieties drove the assembly of these rod-like homoglycopolypeptides into large spherical aggregates.

**Figure 20:**
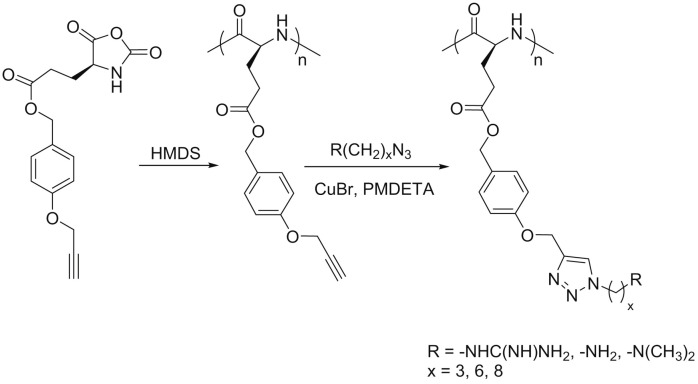
Synthetic route for the preparation of amine/guanidine functionalized polypeptides from poly(γ-(4-propargyloxybenzyl)-l-glutamic acid). Reproduced with permission from reference [89].

Recently, Cheng and co-workers [89] introduced poly(γ-(4-propargyloxybenzyl)-l-glutamic acid) (PPOBLG), which was modified with different amine and guanidine functions via CuAAC for the evaluation of their use in gene delivery purposes. As shown in Figure 20, PPOBLG was polymerized first via ROP of POB-l-Glu-NCA initiated by HMDS. Then, the polymer was post-functionalized in the side-chain via the azide-alkyne Huisgen cycloaddition. HMDS allowed a controlled ROP, yielding well-defined polypeptides with narrow molecular weight distributions (MWDs, ~1.05) and desired degree of polymerization. Owing to the high efficiency of the “click” chemistry, the conjugation efficiencies of amine- or guanidine-containing side chains reached over 99%.

**Figure 21:**
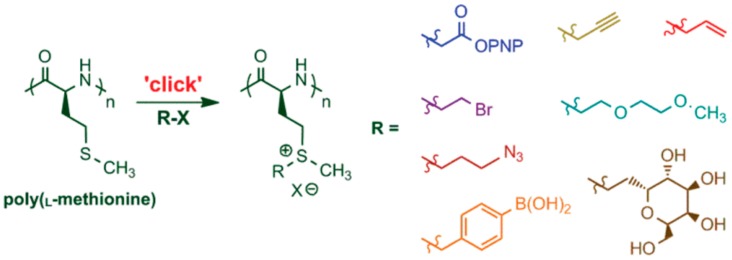
Preparation of Multifunctional and Multireactive Polypeptides via Methionine Alkylation. Reproduced with permission from reference [90].

Deming et al. [90] demonstrated that the thioether group in poly(l-methionine) can be directly functionalized by alkylation with various alkylation reagents under mild conditions, allowing facile preparation of water-soluble polypeptides (Figure 21). They later extend and improved this approach and reported a methodology for the efficient alkylation of methionine residues using epoxides as a general strategy to introduce a wide range of functional groups onto polypeptides [91]. Interestingly, the use of a spacer between epoxide and functional groups further allowed the addition of sterically demanding functionalities. Contrary to other methods to alkylate methionine residues, epoxide alkylations allowed the reactions to be conducted in wet protic media and gave sulfonium products that were stable against dealkylation. These functionalizations were chemoselective, utilized stable and readily available epoxides, and allowed the facile incorporation of an unprecedented range of functional groups onto simple polypeptides using stable linkages. 

**Figure 22:**
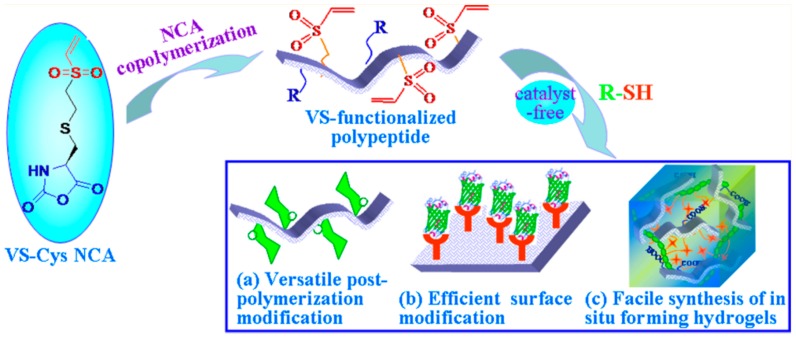
Poly(vinyl sulfone-l-cysteine) modified by Michael addition. Reproduced with permission from reference [92].

The group of Zhong [92] described the synthesis of vinyl sulfone-substituted l-cysteine *N*-carboxyanhydride (VSCys-NCA) monomer to afford a novel and versatile family of vinyl sulfone (VS)-functionalized polypeptides. These polypeptides offered a facile access to functional polypeptide-based materials including glycopolypeptides, functional polypeptide coatings, and in situ forming polypeptide hydrogels through Michael-type addition chemistry under mild conditions (Figure 22). The copolymerization of γ-benzyl-l-glutamate NCA (BLG-NCA), *N*-benzyloxycarbonyl-l-lysine NCA (ZLL-NCA), or l-leucine NCA (Leu-NCA) with VSCys-NCA using 1,1,1-trimethyl-*N*-2-propenylsilanamine (TMPS) as an initiator proceeded smoothly in DMF at 40 °C, yielding P(BLG-*co*-VSCys), P(ZLL-*co*-VSCys), or P(Leu-*co*-VSCys) with defined functionalities, controlled molecular weights, and moderate polydispersities below 1.5. The acidic deprotection of P(BLG-*co*-VSCys) and P(ZLL-*co*-VSCys) furnished water-soluble VS-functionalized poly(l-glutamic acid) (P(Glu-*co*-VSCys)) and VS-functionalized poly(l-lysine) (P(LL-*co*-VSCys)), respectively. These VS-functionalized polypeptides were amenable to direct, efficient, and selective postpolymerization modification with varying thiol-containing molecules such as 2-mercaptoethanol, 2-mercaptoethylamine hydrochloride, l-cysteine, and thiolated galactose providing functional polypeptides containing pendant hydroxyl, amine, amino acid, and saccharide, respectively. Due to the absence of radicals, high temperatures or UV radiation, the conditions for the Michael addition were much milder, potentially reducing side reactions (like radical recombination/disproportionation). 

Finally, two recent examples were reported by the groups of Tang [93] and Deming [94]. Halogenide NCAs, i.e., γ-chloropropyl side chain functional groups were synthesized by Tang et al. [93] that polymerized γ-4-chloromethylbenzyl-l-glutamate NCA and used the chloride to introduce 1-alkylimidazolium into the side chain. The thermo-responsive behavior of the resulting polycations could be tuned by exchange of the counter-ion. Deming [94] used the nucleophilic substitution of bromide to synthesize poly(l-phosphorylcholine homoserine). Interestingly, both the removal of the benzyl protective groups and the amination was performed in one step. In contrast, this method was found to be not suitable for poly(l-phosphorylcholine serine) due to β-elimination of the serine, followed by chain degradation.

#### 4.1.2. Synthesis and Post-Modification of Random Copolypeptides

While it is true that natural proteins present an extremely complex structure as a result of the combination of up to 20 different R-amino acids, synthetic random and statistical copolypeptides can, at least to some extent, structurally mimic the 3D structure found in proteins. Random copolypeptides are typically prepared by simultaneous copolymerization of two or more different NCAs. The copolymerization step is clearly affected by different parameters including the type of initiator or the solvent employed. Nevertheless, once these parameters have been optimized, the knowledge of the relative reactivity of the different NCA monomers employed is crucial to determine the amino acid sequence distribution. Different techniques have been employed for this purpose including ^1^H-, ^15^N-, and ^13^C-CP/MAS NMR techniques. 

For instance, Wamsley et al. [95] reported the synthesis of different binary copolypeptides of β-benzyl aspartate-NCA (BLA), leucine-NCA (L-Leu) and valine-NCA (L-Val), i.e., combinations of L-Leu with BLA, BLA with L-Val, and L-Leu with L-Val were also prepared, parallel their reactivity ratios were obtained through three methods, i.e., Fineman-Ross, Kelen-Tüdos graphical methods and the nonlinear least-squares curve fitting method. On the basis of these analyses, they found that the reactivity ratios varied as follows: β-benzyl aspartate-NCA > leucine-NCA > valine-NCA. Moreover, they explored also the fabrication of ternary copolymers. According to their findings, although the complexity increased from binary polymerizations to ternary polymerizations, the addition of a third NCA in the polymerization of poly(leucine-β-Bzl-aspartate-valine) did not adversely affect the randomness of the reaction, as determined from binary copolymerizations. The randomness of the terpolymer was confirmed because there was no difference between the terpolymer compositions determined experimentally and those predicted with the Alfrey–Goldfinger equations on the basis of the reactivity ratios obtained from binary copolymerizations.

A large number of examples have been reported for the preparation of copolymers and terpolymers combining two or more different NCAs. Examples of these copolymers include: γ-benzyl l-glutamate and l-methionine [96], γ-benzyl l-glutamate and l-valine [97,98], Glycine and Alanine [99], d,l-leucine and d,l-valine [100,101], *N*^ε^-carbobenzoxy-l-lysine and γ-benzyl-l-aspartate, [102] *O*-acetyl-l-tyrosine with l-valine and glycine, [103] l-alanine and l-valine [104] *N*^ε^-carbobenzoxy l-lysine and l-valine [105], l-Alanine and sarcosine [106], γ-methylglutamate and l-leucine [107], *N*^ε^-carbobenzoxy l-lysine and *O*,*O*′-dicarbobenzoxy-l-dihydroxyphenylalanine [108,109], *O*-phospho-l-threonine and l-aspartic acid by the corresponding phenyl- or benzyl-protected NCAs [110] l-Lysine and one of the hydrophobic amino acids l-Leucine, l-Phenylalanine, l-Isoleucine, l-Valine, or l-Alanine [111], γ-benzyl l-glutamate and *N*^ε^-carbobenzoxy l-lysine NCA with lysine NCAs carrying labile protective groups such as *N*^ε^-trifluoroacetyl-(TFA), *N*^ε^-(*tert*-butoxycarbonyl)-(Boc), *N*^ε^-(9-fluorenylmethoxycarbonyl)-(Fmoc), and *N*^ε^-(6-nitroveratryloxycarbonyl)-(Nvoc), γ-benzyl-l-glutamate and *N*^ε^-carbobenzoxy-l-lysine NCA [32,107], Proline and Alanine [112], γ-methyl-l-glutamate, l-glutamic acid [113,114], random terpolymers, such as of glycine, l-leucine, and l-valine [115] l-Leucine, l-Valine, and β-benzyl l-aspartate [95], l-Glutamic acid, l-lysine, and l-Tyrosine [116].

An alternative approach for the fabrication of random copolypeptide was described by Higuchi et al. [113,114] based on the protection-deprotection chemistry. Their approach involved the homopolymerization of a single type of NCA bearing side protective groups. Controlled partial deprotection leads a “pseudo” copolymer that combined protected (hydrophobic) and deprotected (hydrophilic) side functional groups. In particular, the authors, firstly polymerized γ-methyl-l-glutamate-NCA in 1,2-dichloroethane employing *n*-hexylamine. Secondly, the final homopolymer was dissolved in a mixture of methanol and isopropanol and treated with aqueous NaOH for 10 h, followed by treatment with trifluoroacetic acid to afford the random copolypeptide poly[(γ-methyl-l-glutamate)-*co*-(l-glutamic acid)] (poly(MLG)-*co*-(LGA)). NMR analysis revealed that 30% of the monomer units had been transformed to glutamic acid.

**Figure 23:**
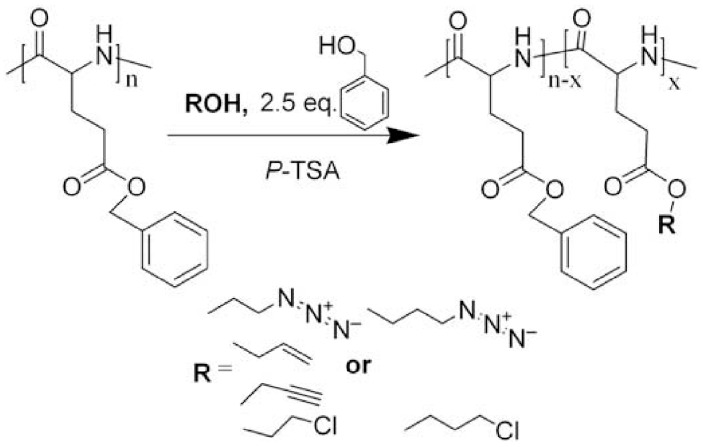
Synthesis of functional PBLGs through ester exchange reactions. Reproduced with permission from reference [117].

Side-chain modification of copolypeptides has been also a widely employed strategy to introduce novel functionalities on the polypeptide chain. For instance, four types of functional poly(γ-benzyl-l-glutamate) (PBLG) copolymers containing chloro, azido, allyl or propargyl groups on the side chains were synthesized through ester exchange reactions of PBLG with functional alcohols without any protection and de-protection process. Guo et al. [117] reported the hydrolysis of PBLG, (which was found during the ester exchange reaction under low ratios of alcohol to the repeat units of PBLG) was successfully depressed by addition of a certain amount of benzyl alcohol to the reaction system (Figure 23). Click chemistry reactions of the azidized or propargylated copolymers, thiol-ene reaction of the allyllated copolymer were taken successfully, indicating that the functional groups on the copolymers were still reactive. 

**Figure 24:**
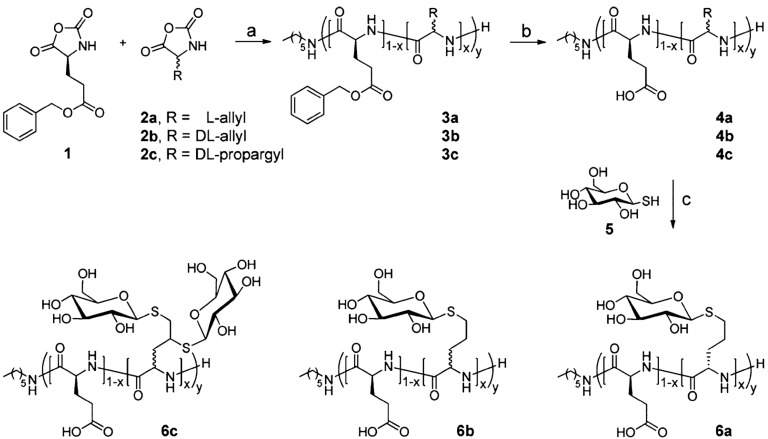
Synthesis of Glucocopolypeptides by NCA Copolymerization and Thiol-Ene/Yne Photochemistry. Reagents and conditions: (a) 1-hexylamine, DMF, 25 °C, 7 days; (b) MSA/anisole/TFA, 0−20 °C, 38 min; (c) Irgacure 2959, hν, 0.1 M aqueous acetate buffer, 25 °C, 12 h. Reproduced with permission from reference [42].

A similar strategy was described by Krannig and Schlaad [42] for the synthesis of copolypeptides of l-glutamate and glucosylated l-/dL-allyl- or dL-propargylglycine. The latter were prepared by ring-opening polymerization and thiol-ene/yne photochemistry in aqueous solution (Figure 24). As a result, the authors reported an interesting strategy to introduce using mild conditions for the introduction of the sugar units (glucose) in the final step. Interestingly, the secondary conformation transition observed in poly(l-glutamate) remained and both glucosylated and non-glucosylated samples adopted a random-coil conformation in neutral and basic media and an α-helical conformation in acidic media. Nevertheless, the helical content depended on the number and configuration of allyl-/propargylglycine units. 

**Figure 25:**
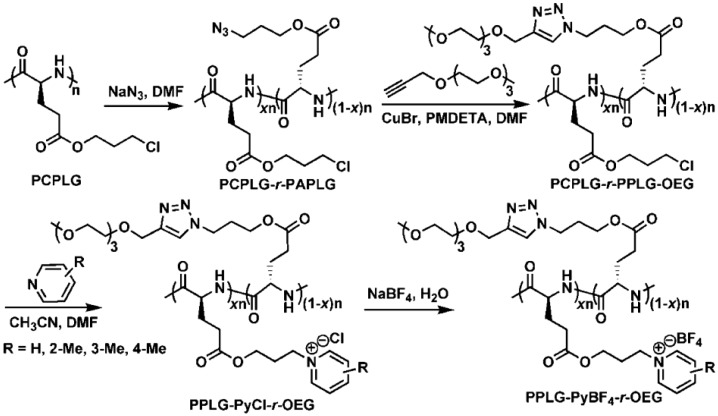
Synthetic Route of PPLG-PyBF_4_-r-OEG. Reproduced with permission from reference [118].

A recent example was reported by Zhu et al. [118] describing the synthesis of random copolypeptides bearing oligo ethylene glycol (OEG) and pyridinium tetrafluoroborate (PyBF_4_) pendant side chains. The synthetic strategy, depicted in Figure 25, required a four-step postpolymerization from poly(γ-3-chloropropyl-l-glutamate) (PCPLG) which was prepared according to reported procedures. Poly(γ-3-chloropropyl-l-glutamate)-random-poly(γ-3-azidopropyl-l-glutamate)s (PCPLG-r-PAPLGs) with constant main-chain length and various molar content of chloro groups (x) were obtained by reacting PCPLG with sodium azide (NaN_3_) in DMF at 60 °C. Different x values were achieved by regulating the reaction time. Poly(γ-3-chloropropyl-l-glutamate)-random-poly(γ-propyl-l-glutamate)-*graft*-(oligo-ethylene glycol)s (PCPLG-r-PPLG-OEGs) were synthesized by copper-mediated [2 + 3] alkyne-azide 1,3-dipolar cycloaddition between propargyl functionalized oligo-ethylene glycol (Pr-OEG) and PCPLG-r-PAPLG.

### 4.2. Strategies for the Preparation of Block Copolymers Using NCAs

Despite few examples devoted to the functionality of random synthetic copolypeptides, applications of the latter are limited mainly due to the limited control over the random amino acid NCA polymerization [107]. As a result, block copolymers with precise peptide segment sequences and controlled molecular weight may improve the drawbacks observed in random and statistical copolypeptides. 

Block copolymers containing polypeptide blocks have been typically classified into two main groups depending on the block composition. The first group includes those copolypeptide structures, where both blocks are polypeptides. In the second group, block copolymer structures are formed by a polypeptide combined with other non-peptide polymer block thus forming hybrid structures. In the next subsections selected examples from both, block copolypeptides and hybrid copolymers will be presented. 

#### 4.2.1. Block Copolymers Comprising Exclusively Polypeptides: Block Copolypeptides

Block copolypeptides are, in general, prepared by sequential addition of different NCAs, either to amine or transition metal complex initiators. Although triblock copolypeptides, and other more complex linear copolypeptides, have been prepared, the synthesis of diblock copolypeptides has been the center of multiple studies. 

**Figure 26:**
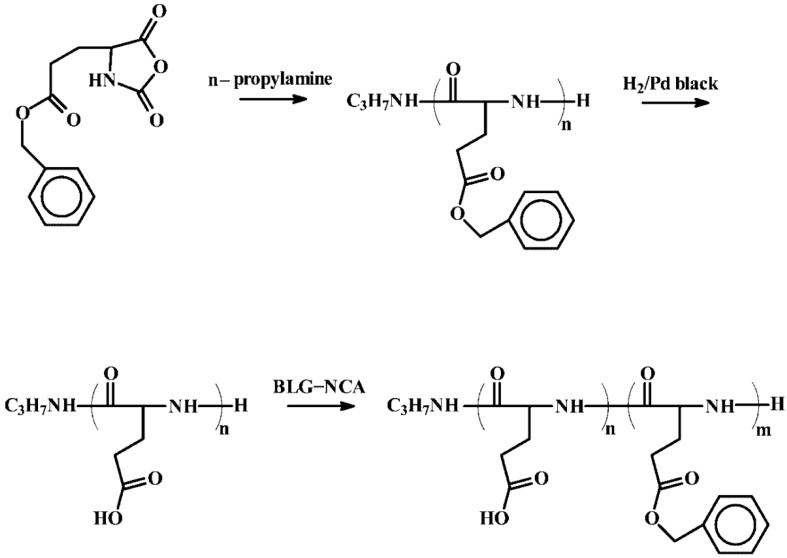
Synthesis of poly[(γ-benzyl-l-glutamate)-*b*-(l-glutamic acid)] proposed by Higashi et al. [121].

The general strategy to produce these block copolymers involves the sequential addition of monomers, assuming complete conversion of the previous monomer [119,120]. An illustrative example of this methodology was reported by Aoi et al. [119]. They used *n*-hexylamine as initiator and polymerized first *O*-(Tetra-*O*-acetyl-d-glucopyranosyl)-l-serine-NCA followed by Ala-NCA to synthesize the block copolypeptide. SEC analysis confirmed the complete consumption of the first block. Both blocks had low molecular weight, whereas the molecular weight distribution (SEC) were very narrow. An identical strategy was followed by Higashi et al. [121] for the preparation of poly[(BLG)-*b*-(LGA)] block copolymers. As depicted in Figure 26, BLG-NCA was polymerized first using *N*-propylamine as an initiator. After precipitation of the PBLG block in diethyl ether and benzyl protective group removal by catalytic hydrogenolysis (Pd/H_2_), a second polymerization step was carried out using again protected BLG-NCA. Also, Guillermain et al. [120] employed a two polymerization step approach to fabricate amphiphilic block copolypeptides of poly(*N*^ε^-trifluoroacetyl-l-lysine) and a poly(l-lysine) block bearing liquid crystalline side groups. These liquid crystalline blocks include poly[11-(biphenyl-4-carboxamido)undecanamido-l-lysine] and poly(*N*^ε^-4-phenylbenzamido-l-lysine). 

In order to avoid contamination and thus to reduce the eventual side reactions, Aliferis et al. [76,122,123] resort to the use of high vacuum techniques (HVT) for the sequential polymerization of different NCAs. For their studies, they also employed primary amines as initiators but in contrast to previously employed methodologies, HVT ensures that all the reagents and the level of impurities of the reaction environment were rather low in all the reaction steps and living nature of a polymerization method is assumed. Following this methodology, the authors reported the preparation of well-defined block copolypeptides including PBLG-PZLL, PBLG-PTYR, PBLG-PGLY, PZLL-PBLG, and PBLG-PLEU. The molecular weights of the syntheses above mentioned, were monitored by SEC and membrane osmometry. The results confirmed the low-level of impurities, avoiding termination reaction and promoting the living polymerization of the NCAs.

**Figure 27:**
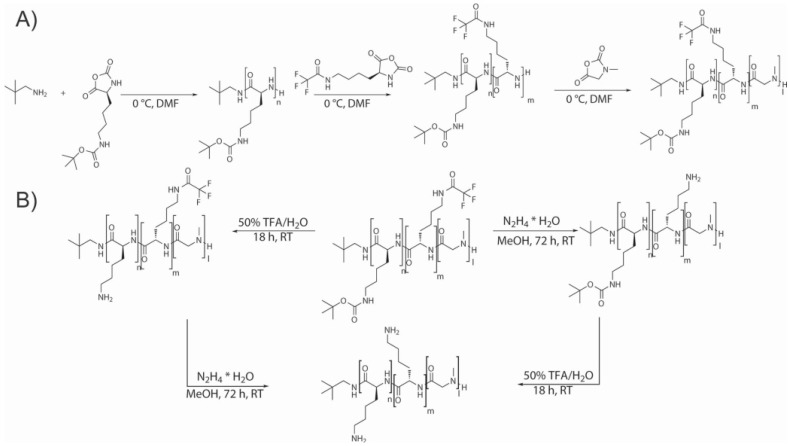
(**A**) Synthesis of PLys(Boc)-PLys(TFA)-PSar triblock copolymers by sequential ring-opening polymerization followed by (**B**) Block specific deprotection of the PLys blocks. Reproduced with permission from reference [124].

Recently, the synthesis of triblock copolymers based on polysarcosine, poly-*N*^ε^-t-butyloxycarbonyl-l-lysine, and poly-*N*^ε^-trifluoroacetyl-l-lysine by ring-opening polymerization of the corresponding α-amino acid *N*-carboxyanhydrides (NCAs) was described by Heller et al. [124] (Figure 27). For the synthesis of *N*^ε^-t-butyloxycarbonyl-l-lysine (lysine (Boc)) NCAs, an acid-free method was employed using trimethylsilylchloride/triethylamine as hydrochloric acid (HCl) scavengers. This approach enabled the synthesis of lysine (Boc) NCA of high purity in high yields. For triblock copolypeptides, the degree of polymerization of the polysarcosine block was varied between 200 and 600; poly-*N*^ε^-t-butyloxycarbonyl-l-lysine and poly-*N*-ε-t-trifluoroacetyl-l-lysine blocks were designed to have a Xn the range of 10–50. The polypeptide-polypeptoid hybrids (polypept(o)ides) could be synthesized with precise control of molecular weight, high end group integrity, and dispersities indices between 1.1 and 1.2. But more important, the use of tertbutyloxycarbonyl- and trifluoroacetyl-protecting groups permitted the selective, orthogonal deprotection of both blocks, which enabled further post-polymerization modification reactions in a block-selective manner. Therefore, this synthetic approach provided a versatile pathway to triblock copolypept(o)ides, in which functionalities can be separated in specific blocks.

**Figure 28:**
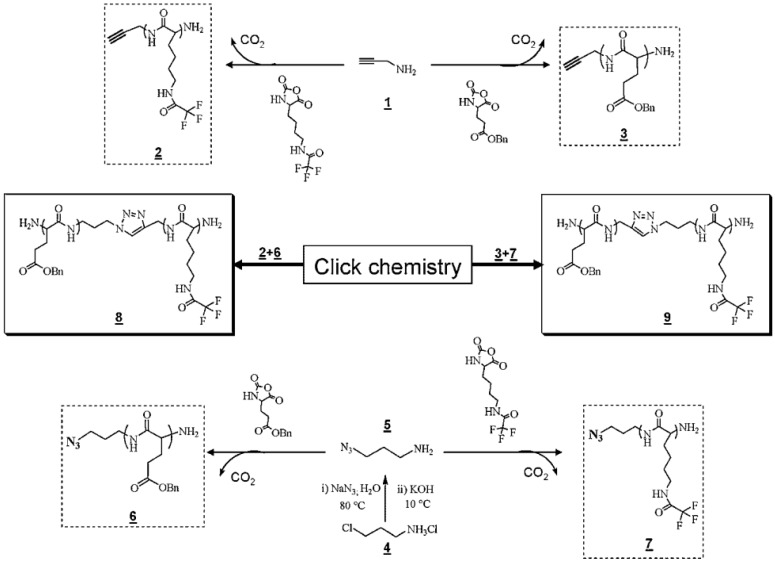
Synthesis of PBLGlu-*b*-PTFALys diblock copolymers by click chemistry as reported by Agut et al. [125].

In addition to a two or multi-step consecutive strategy, in a recent work, Agut et al. [125] combined the ring-opening polymerization (ROP) of *N*-carboxyanhydrides (NCAs) from α-ω-functionalized initiators with the Huisgen 1,3 dipolar cycloaddition (click chemistry). As depicted in Figure 28, poly(γ-benzyl-l-glutamate) (PBLGlu) and poly(trifluoroacetyl-l-Lysine) (PTFALys) functionalized with either an azide or an alkyne functional group in α-position, was first synthesized by ROP of the corresponding NCA at room temperature in DMF as solvent, using appropriate ω-amino-containing α-alkyne and α-azido difunctional initiators. In order to couple the homopolypeptide block, copper(I)-catalyzed coupling reactions of α-azido-PBLGlu with the α-alkyne-PTFALys, on the one hand, and of the α-alkyne-PBLGlu with the α-azido-PTFALys, on the other hand were carried out. As a result, the targeted PBLGlu-*b*-PTFALys diblock copolypeptides possessing a triazole group in between the two blocks were obtained. Nevertheless, the block copolypeptides required further purification by selective extraction with chloroform.

Triblock copolypeptides of the ABA type have been also prepared using identical strategies as those depicted above. However, the fabrication of triblock structures required the use of difunctional amine initiators. The most commonly employed initiator is 1,6-hexamethylenediamine. One of the pioneer works attempting to fabricate triblock copolypeptides was reported by Minoura et al. [126,127]. This group reported the fabrication of poly[(BLG)-*b*-(L-leucine)-*b*-(γ-BLG)] and their corresponding poly[(LGA)-*b*-(L-leucine)-*b*-(LGA)] triblock copolypeptide. However, the protocol followed required further purification of the reagents (solvent, monomers, etc.) since the presence of homopolypeptides was also observed.

A significant improvement was reported by V. Breedveld et al. [128,129] They employed a complex structure, i.e., Co(PMe_3_)_4_ as an initiator for the preparation of poly[(*N*^ε^-carbobenzoxy l-lysine)-*b*-(L-leucine)-*b*-(*N*^ε^-carbobenzoxy l-lysine)]. The complex-mediated the polymerization, permitted high conversions while maintaining a relatively narrow molecular weight distributions. 

Table 3 shows some common examples (non-exhaustive list) of block copolypeptides reported in the literature.

#### 4.2.2. Preparation of Linear Hybrid-Polypeptide Block Copolymers

Hybrid copolymers bearing polypeptide block have been prepared to combine several polymerization techniques for the synthesis of the non-polypeptide block with ROP NCAs. In this section, we will provide selected recent examples of hybrid polypeptides based on the methodology employed for their preparation. However, before describing their synthesis, few aspects of the chain end modification required for their fabrication will be analyzed.

##### Chain-End Modification of Polypeptides

The modification of chain-ends with particular functional groups is, in general, a prerequisite for the fabrication of linear hybrid block copolypeptides. In order to fabricate polypeptides with the presence of the desired functional groups at the chain ends two main strategies have been proposed. 

(a) Using an Initiator that Contains the Desired Functional Group

**Figure 29:**
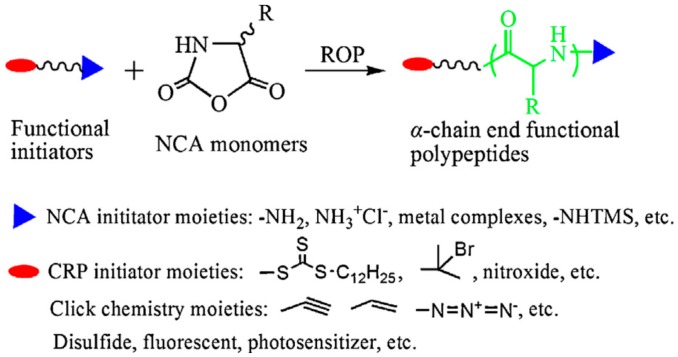
Alternatives to fabricate α-chain end modified polypeptides using functional initiators. Reproduced with permission from reference [31].

On the one hand, different groups resorted to the use of functional initiators. As depicted in Figure 29, depending on the initiator employed not only functional groups in the α-position can be obtained but also in the ω-position. The ω-position provides amines, metal complexes and NHTMS functional groups for further modification. The α-position, more versatile in terms of chemical groups, can be decorated with functional such functional groups as initiators for controlled radical polymerization (CRP), functional initiators containing an azido, alkyne, or alkene group to combine NCA polymerization with click chemistry or functional initiators containing disulfide, fluorophore or photosensitizer. In particular, the functionalization of the α-position using hetero-functional initiator strategy presents several advantages. As explained by Chao et al. [31] this strategy: (1) avoid tedious synthesis and incomplete transformation of polymer chain ends for CRP; (2) permits the combination of various polymerization techniques (ROP/ATRP, ROP/RAFT, ROP/NMP); and (3) it is possibly conduct dual polymerization in one pot without intermediate purification steps.

**Figure 30:**
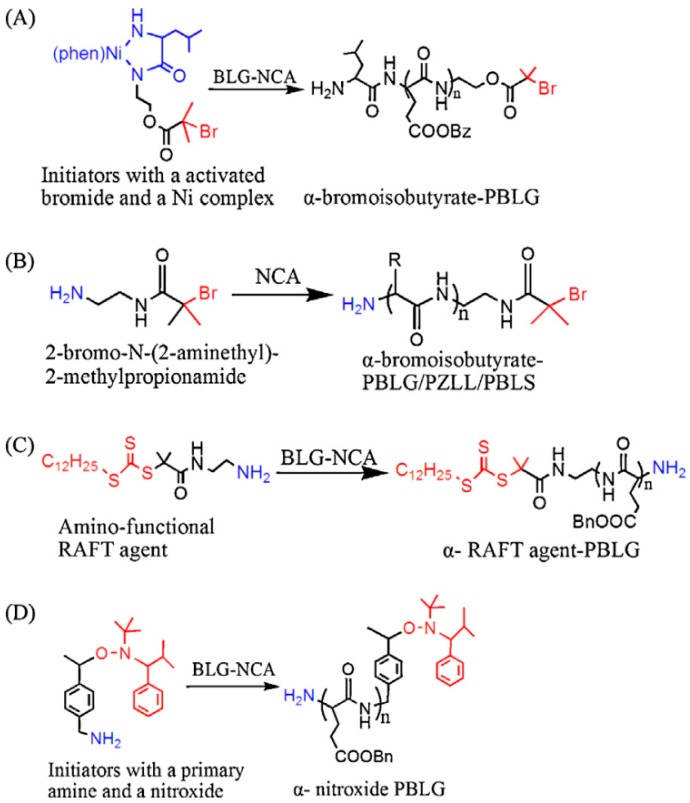
Dual initiators capable of promoting NCA polymerization and controlled radical polymerization. (**A**,**B**) Dual initiators for NCA polymerization and atom transfer radical polymerization (ATRP); (**C**) dual initiators for NCA polymerization and reversible addition-fragmentation chain-transfer (RAFT) polymerization; and (**D**) dual initiators for NCA polymerization and nitroxide-mediated radical polymerization (NMP). Reproduced with permission from reference [31].

**Figure 31:**
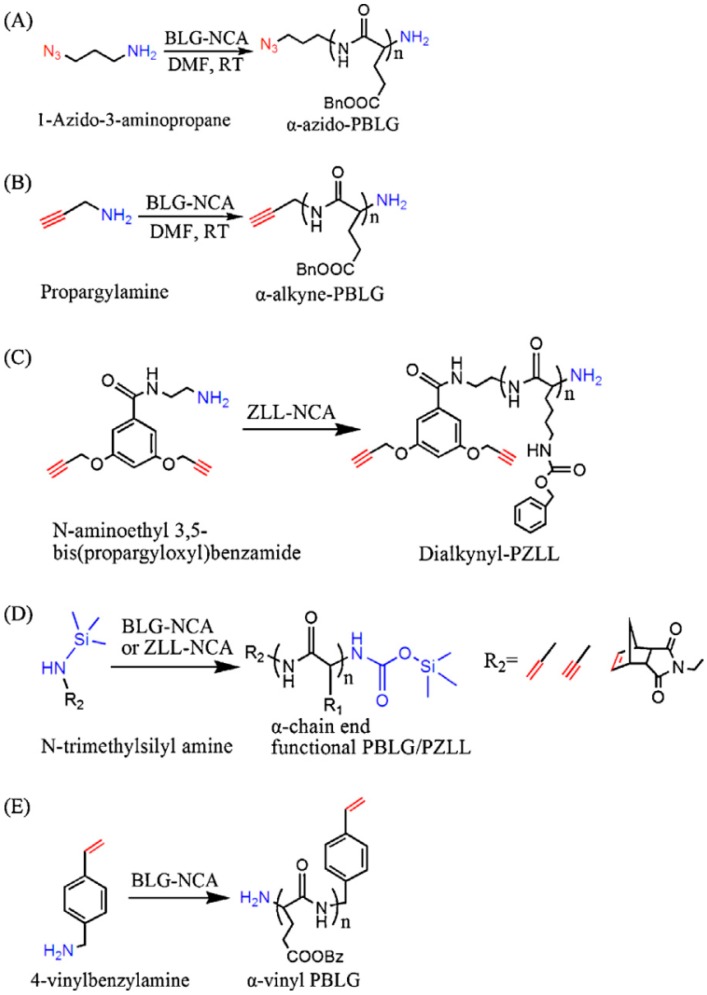
Synthesis of α-chain end functionalized polypeptides from initiators containing azido groups (**A**), alkyne groups (**B**–**D**), or alkene groups (**D**, **E**). Reproduced with permission from reference [31].

In the review published by Chao et al. [31] a thorough description of the end-modification alternatives of linear polypeptides is presented. They summarized the most extensively employed approaches and classify them into two main families. On the one hand, as illustrated in Figure 30, dual initiators capable of promoting NCA polymerization and controlled radical polymerization. These include dual initiators for NCA polymerization and ATRP polymerization (A, B); dual initiators for NCA polymerization and RAFT polymerization (C); and dual initiators for NCA polymerization and NMP polymerization (D). On the other hand, Figure 31, those approaches that involve the synthesis of α-chain end functionalized polypeptides from initiators containing azido groups (A), alkyne groups (B, C, D), or alkene groups (D, E).

(b) Chemical Modification of the End Functional Groups.

As has been mentioned above, primary amine groups are generally obtained upon the ROP NCAs. Therefore, these can be chemically modified to introduce other alternative functionalities. This strategy has been employed to a much lower extent. One of the few examples using this strategy was reported by Deming et al. [142]. They reported a straightforward methodology for preparation of *N*-terminal functionalized polypeptide block copolymers that complemented the established technique of using amino-functionalized polymers to prepare C-terminal functionalized polypeptides. For that purpose, they hypothesized and confirmed later that isocyanates, or other electrophiles, added to polymerizations, were able to react with the amido-metallacycle propagating species resulting in *N*-terminal capping of the chains through the formation of stable urea linkages.

Based on this procedure Kros et al. [143], described the synthesis and self-assembly of hybrid block copolymers composed of a poly(γ-benzyl-l-glutamate) block (PBLG) and two different polyisocyanide blocks, namely, poly((*S*)-α-methylbenzyl isocyanide) (PMBI) and poly(l-isocyanoalanyl-l-alanine methyl ester) (l,lPIAA). The diblock copolymers were synthesized by the nickel-catalyzed living polymerization of γ-benzyl-l-glutamate *N*-carboxyanhydride (BnGlu NCA) followed by the addition of (S)-α-methylbenzyl isocyanide (MBI) or l-isocyanoalanyl-l-alanine methyl ester to the reaction mixture.

Functional polypeptides have also been prepared by conjugating functional molecules to ω-amino polypeptides. For instance, Fluorescein isothiocyanate (FITC) was conjugated to the ω-amino end of mPEG-SS-PLeu in the presence of triethylamine to yield FITC-labeled polypeptide hybrid copolymer [144]. These FITC-labeled copolymers self-assembled into micelles with a fluorescent core, that facilitated the observation of micelle internalization and trafficking in the cells by confocal laser scanning microscopy (CLSM). 

##### Fabrication of Hybrid Block Polypeptides 

An exhaustive discussion of the strategies reported to fabricate this type of block copolymers has been listed by Hadjichristidis et al. [12] and Deming et al. [3]. In their reports, they described the synthesis of polypeptide hybrid block copolymers according to the initiators used for the ROP NCAs: (a) amines; (b) transition metal complexes; and (c) amine salts. 

Herein, we aim to actualize that review and, offer a novel classification and organization of the hybrid block copolypeptides depending on the type of polymerization involved in the fabrication of the non-polypeptide block. As a result, the most extended strategies can be summarized as follows.

(a) Use of Commercially Available Macroinitiators End-Functionalized with a Primary Amine.

Commercially available end-functionalized polymers carrying end-terminal amine groups (either at one or at both chain ends) have been extensively used as initiators in the polymerization of NCAs for the fabrication of hybrid block copolypeptides. Among the commercial amine terminated homopolymers poly(ethylene oxide) (PEO) is by far the most extensively employed. Amine-terminated PEOs are available in a wide variety of molecular weights with narrow distributions, parallel it can be employed to form amphiphilic, double hydrophilic, and rod-coil block copolymers in one single step. Examples of hybrid block copolymers prepared using PEO are included in Table 4.

(b) Use of Either Conventional or Controlled Radical Polymerization Techniques. 

Radical polymerization is nowadays one of the most extensively employed polymerization reactions for the fabrication of polymers from many different monomers. Radical polymerization has been also explored by Cheon et al. [145] for the fabrication of amine-terminated poly(*N*-isopropylacrylamide) in the presence of 2-amino-ethanthiol hydrochloride as chain transfer agent. These amino-macroinitiators were, in turn, used for the synthesis of hybrid block copolymers with PBLG. Conventional radical polymerization has, however, serious limitations in terms of polymer chain length control, dispersity and even the control over the functional groups present in the polymer. As a result, controlled radical polymerization (CRP) techniques have emerged as alternatives to overcome the limitations mentioned above. Different CRP techniques are currently available, but only three of them have been mainly employed for the fabrication of hybrid block copolypeptides, i.e., atom transfer radical polymerization (ATRP), reversible addition-fragmentation chain-transfer polymerization (RAFT) and nitroxide-mediated radical polymerization (NMP).

One particularly attractive approach for synthesizing hybrid polypeptides is the sequential integration of the ROP of NCAs with other polymerization technologies, such as atom transfer radical polymerization (ATRP), [146,147,148] nitroxide-mediated polymerization (NMP), [149,150] or reversible addition–fragmentation chain-transfer (RAFT) polymerization [151,152].

ATRP was used by Brzezinska et al. [146] for the synthesis of poly(methyl acrylate)-*b*-PBLG. In a first step, the amino end-functionalized poly(methyl acrylate) was prepared by ATRP and then transformed to Ni-macroinitiators for the polymerization of BLG-NCA. This method allows the controlled preparation of polypeptides, control over chain length and without the formation of homopolypeptides contaminants. 

**Figure 32:**
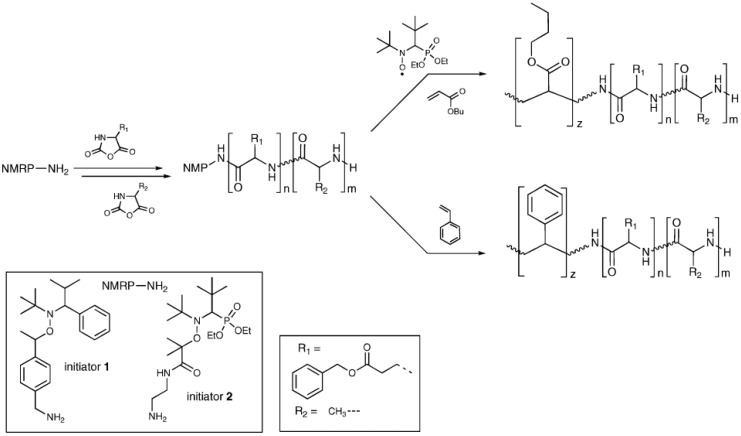
Synthesis of Block Copolymers by Polymerization of *N*-Carboxyanhydrides (NCA) and Styrene or *n*-Butyl Acrylate by Nitroxide-Mediated Polymerization Using Bifunctional Initiators 1 and 2 Reproduced with permission from reference [150,153].

Karatzas et al. [153] used an amine-functionalized TEMPO ((2,2,6,6-Tetramethylpiperidin-1-yl)oxyl) radical (required for the synthesis of poly(*N*-vinylpyrrolidone) (PNVP)) as an initiator for the preparation of the polypeptide block. The amino-group served as the initiator for the polymerization of the corresponding NCAs, leading to well-defined polymers bearing TEMPO end-groups. In a subsequent step, these macroradicals were employed for the NMP of *N*-vinylpyrrolidone (NVP) in the presence of AIBN and acetic anhydride. In particular, the authors selected the PBLG and PZLL peptides to produce PNVP-*b*-PBLG, PNVP-*b*-PZLL, and PNVP-*b*-PBLG-*b*-PZLL triblock copolymers. More recently, Habraken et al. combined NCA polymerization with the nitroxide mediated radical polymerization of poly(*n*-butyl acrylate) (PBA) and polystyrene (PS), using a *N*-*tert*-butyl-*N*-[1-phenyl-2-(methyl-propyl)]nitroxide (TIPNO) and *N*-*tert*-butyl-*N*-[1-diethylphosphono-(2,2-dimethylpropyl)] nitroxide) (SG1)-based bifunctional initiator to create a hybrid block copolymer (Figure 32). The polypeptide block consists of (block) copolymers of poly(l-glutamic acid) embedded with various quantities of l-alanine [150].

Finally, Zhang et al. [151] used RAFT polymerization as controlled polymerization technique to fabricate double-hydrophilic diblock copolypeptides (BCPs), poly(l-glutamic acid)-block-poly(*N*-isopropylacrylamide) (PLGA-*b*-PNIPAM). The diblock copolypeptides were synthesized by a combination of ROP of γ-benzyl-l-glutamate *N*-carboxyanhydrides (BLG-NCA) and reversible addition-fragmentation chain transfer (RAFT) polymerization of *N*-isopropylacrylamide (NiPAM). For this purpose, the authors described a new class of RAFT agents (CTA-2 and CTA-3) with amino-functional groups. Another recent example of hybrid block copolypeptides was reported by Jacobs et al. [152] They synthesize narrow disperse poly(*n*-butyl acrylate), polystyrene, and poly(*N*-isopropyl acrylamide) using reversible addition–fragmentation chain transfer (RAFT) as the synthetic tool. A phthalimidomethyl trithiocarbonate RAFT chain transfer agent was used to prepare well-defined, end-functional polymers, which after deprotection resulted in amine terminal macroinitiators. The subsequent initiating systems could successfully be chain extended with ε-benzyloxycarbonyl-l-lysine or γ-benzyl-l-glutamate as the NCAs to produce a library of polymer–polypeptide conjugates. 

(c) Use of Ring-Opening Polymerization. 

**Figure 33:**
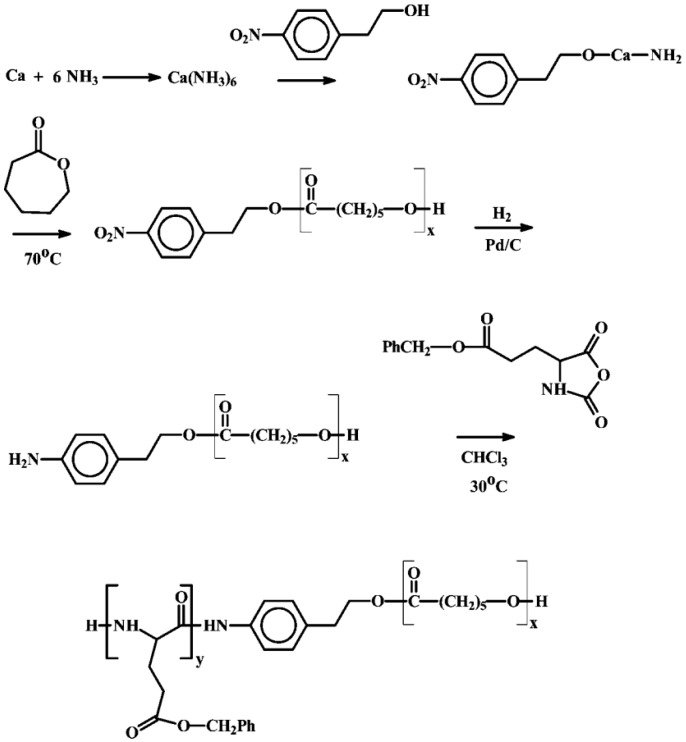
Strategy reported by Rong et al. [156] to prepare a biodegradable, poly(ε-caprolactone)-*b*-poly(γ-benzyl-l-glutamic acid) (PCL-*b*-PBLG) diblock copolymer.

Hybrid block copolymers with aliphatic polyesters are commonly used in some medical related research fields due to their biocompatibility, low immunogenicity, biodegradability and their mechanical strength an interesting class of polymeric materials [154,155]. Among the aliphatic polyesters poly(ε-caprolactone) (PCL) and polylactide (PLA) have received special attention. Similar to the above-mentioned methodologies, the most extended strategy for the fabrication of polyester hybrids involved two steps, i.e., the preparation of either mono- or diamino polyester (A) macroinitiators and ROP of the desired NCA. This strategy has been employed by Rong et al. [156] to prepare a biodegradable, poly(ε-caprolactone)-*b*-poly(γ-benzyl-l-glutamic acid) (PCL-*b*-PBLG) diblock copolymer. An aminophenethoxyl-terminated PCL was prepared first via catalytic hydrogenation of a 4-nitrophenethoxyl-terminated PCL, which, as depicted in Figure 33, was obtained from the polymerization of ε-caprolactone (CL) initiated by amino calcium 4-nitrobenzoxide. In a second step, the authors prepared the polypeptide block by ROP of *N*-carboxyanhydride of γ-benzyl-l-glutamate (BLG-NCA) using the aminophenyl-terminated PCL as a macroinitiator. The aromatic amino groups served as initiating sites for the polymerization of the BLG-NCA to afford PCL-*b*-PBLG. 

Hybrids have also been prepared using PLA macroinitiators and PAla, PPhe, PLeu, PBLG, or poly(benzyl-l-aspartate) as polypeptides [10,157,158]. An illustrative example of this type of block copolymers was reported by Gotsche et al. [157]. They fabricated poly(l-lactide)-*b*-poly(l-aminoacid) block copolymers via polymerization of α-amino acid *N*-carboxyanhydrides with amino-terminated poly(l-lactide)s as macroinitiators. The macroinitiator was obtained by polymerization of (l,l)-lactide with an initiator prepared in-situ from diethylzinc Et_2_Zn and *N*-*tert*-butoxycarbonyl-1-amino-3-propanol, followed by the deprotection of the amino group. 

(d) Use of Anionic Polymerization. 

Similarly to controlled radical polymerization techniques, anionic living polymerizations permit the control over the macromolecular structure and, in particular, over the functional groups present at both chains ends. Anionic polymerization was the methodology employed by Klok and coworkers [159] to prepare a polystyrene oligomer. The termination of the living polymerization was performed with 1-(3-chloropropyl)-2,2,5,5-tetramethyl-1-aza-2,5-disilacyclopentane, the process was followed by an acidolysis of the protective group. The amino-functionalized oligomers were then used to initiate the polymerization of BLG-NCA. Also Schlaad et al. [68,160] also prepared amino end-functionalized PS by anionic polymerization and employed them as macroinitiators for the synthesis of PS-*b*-PZL hybrid block copolymers. The purified copolymers were characterized by very narrow molecular weight distributions. 

(e) Step-Growth Polymerization.

Although this type of polymerization has rarely been employed, step-growth polymerization can also produce telechelic amine macroinitiators. Kong et al. [161] used the Yamamoto coupling polymerization of 2,7-dibromo-9,9-dihexylfluorene, also, they modified the end-terminal groups by end-capping with *N*-(*p*-bromobenzyl)phthalimide. After a deprotection step with hydrazine telechelic, amine macroinitiators were obtained and posteriorly used for the polymerization of BLG-NCA. Due to the nature of the step-growth polymerization, the molecular weight distribution of the triblock copolymers was very broad (DP > 2.0).

(f) Use of Click Chemistry Reactions to Bond Complementary Homopolymers.

In contrast to the above-mentioned strategies that resort to two consecutive polymerization reactions, Lecommandoux et al. [162] prepared polysaccharide-*b*-polypeptide hybrid block copolymers. On the one hand, dextran was exposed to a reductive amination, using propargylamine in acetate buffer (pH = 5.0) in the presence of sodium cyanoborohydride. On the other hand, 1-azido-3-aminopropane was employed as a functional initiator for the polymerization of BLG-NCA. Finally, both polysaccharide and polypeptide blocks were coupled in the presence of CuBr, in DMSO, and the ligand pentamethyldiethylenetriamine, PMDETA, at room temperature. An excess of dextran was employed for the quantitative reaction of the functionalized PBLG chain, which was removed later by dialysis against water. More recently, the same group prepared well-defined block copolymers composed of a rigid poly(γ-benzyl-l-glutamate) (PBLG) sequence and a poly[2-(dimethylamino)ethyl methacrylate] (PDMAEMA) block were synthesized by Huisgen’s 1,3-dipolar cycloaddition (click chemistry) from homopolymers containing azide and alkyne functionalities [163]. These functional groups were introduced in the α-position of both PBLG and PDMAEMA precursors using appropriate α-ω-functionalized initiators to trigger the living/controlled polymerization of the corresponding monomers. Both α-alkyne- and α-azido-PBLGs were synthesized by ring-opening polymerization of γ-benzyl-l-glutamate *N*-carboxyanhydride at room temperature from amino-containing α-alkyne and α-azide difunctional initiators, using dimethylformamide as solvent. As for the case of the α-alkyne-PDMAEMA and α-azido-PDMAEMA, they were obtained by copper-mediated atom transfer radical polymerization of 2-(dimethylamino)ethyl methacrylate at 60 °C in tetrahydrofuran as solvent. The copper (I)-catalyzed 1,3-dipolar cycloaddition coupling reactions of the α-azido-PBLG with the α-alkyne-PDMAEMA, in the one hand, and of the α-alkyne-PBLG with the α-azido-PDMAEMA, on the other hand were performed in DMF and afforded the targeted PBLG-*b*-PDMAEMA diblock copolymers. 

#### 4.2.3. Post-Modification of Block Copolypeptides

The last alternative to fabricate novel block copolypeptides consists on the post-modification of the block copolypeptide obtained by any of the methodologies described above. Engler et al. [87,88] employed this approach to prepare a series of pH responsive synthetic polypeptides based on an *N*-carboxyanhydride ring opening polymerization combined with click chemistry modification step. Poly(γ-propargyl l-glutamate) (PPLG) homopolymers and poly(ethylene glycol-*b*-γ-propargyl l-glutamate) (PEG-*b*-PPLG) block copolymers were substituted with various amine moieties that range in p*K*a and hydrophobicity, providing the basis for a library of new synthetic structures that can be tuned for specific interactions and responsive behaviors. These amine-functionalized polypeptides had the ability to change solubility, or reversibly self-assemble into micelles with changes in the degree of ionization; they also adopt an α-helical structure at biologically relevant pHs. 

## 5. Strategies to Fabricate Cyclic Polypeptides

As explained in previous reviews [12], the formation of cyclic structures is basically directed by the type of initiator employed. On the one hand, the use of protic nucleophile, (e.g., *n*-hexylamine) or even alcohols, generally produce linear polypeptides. On the contrary, the use secondary or tertiary amines as initiators are more adapted for the synthesis of cyclic polypeptides. The influence of the reaction conditions on the extent of cyclization was studied in more detail by Kricheldorf et al. [178] by MALDI-TOF mass spectroscopy. As a result, the authors proposed three main mechanisms to form cyclic polypeptides. 

**Figure 34:**
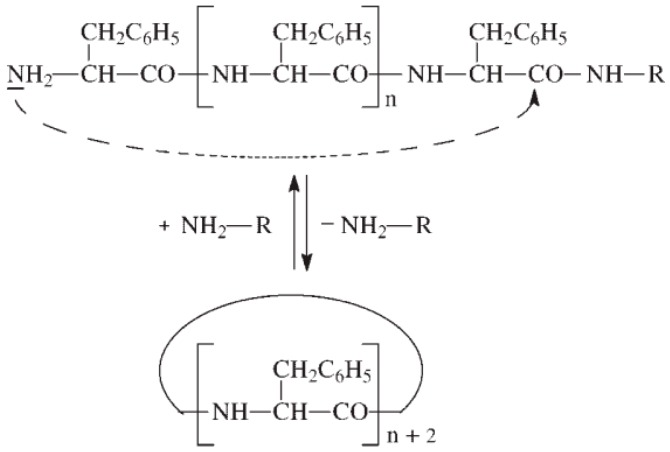
Formation of cyclic oligo-/polypeptides by “back-biting”.

The formation of cyclic oligopeptides occurs when the amino end groups can cleave peptide bonds or amide end groups, so that a so-called “back-biting” equilibration occurs (Figure 34). According to the authors, such a “back-biting” reaction is not observed for polypeptides at 20 °C but occurs above 220 °C in molten polyamides. Moreover, such reaction should also take place in dioxane and sulfolane. 

**Figure 35:**
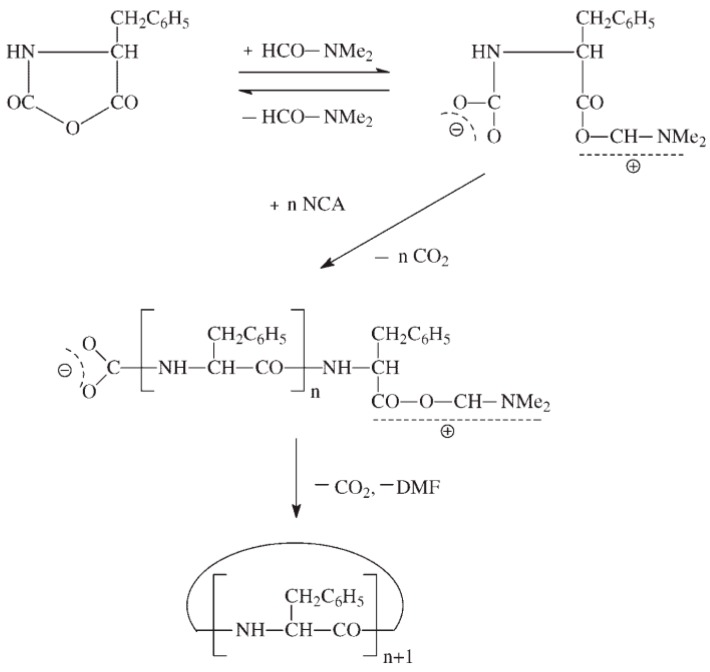
Formation of cyclic oligo-/polypeptides via zwitterionic polymerization.

**Figure 36:**
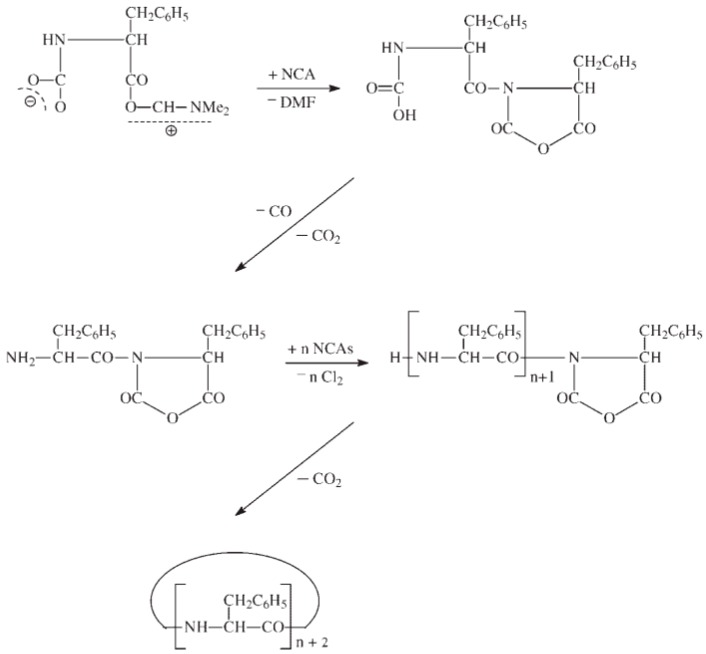
Formation of cyclic oligo-/polypeptides via *N*-acyl NCA end groups.

The other two cyclization mechanisms share the same initiation process, i.e., zwitterionic polymerization. DMF and NMP are the solvents with the highest nucleophilicity and basicity, and thus, prone to initiate a polymerization via zwitterions (Figure 35 and Figure 36). For instance, in the case of d,l-Phe-NCA the spontaneous polymerization occurred at 20 °C and cyclic polypeptides were the largely prevailing products in DMF and in NMP according to the mechanism proposed in Figure 35. Based on the MALDI-TOF results, m.s. ≥ 95 of the reaction product consisted of cycles.

Finally, the third mechanism proposed involves the formation of cycles via *N*-acyl NCA end groups. The zwitterionic polymerization can, in principle, follow two different kinetics courses. The chain growth consisted either of ring-opening polymerization involving a chain growth kinetics or polycondensation step involving step growth kinetics. Both kinetics courses produce identical chains and end groups, but the molecular weight distributions should be different. The polymerization of l-Phe NCA can be explained by this mechanism as outlined in Figure 36. Oligo- and poly(l-phenylalanine) possess a low solubility in all non-acidic organic solvent and rapidly precipitate from the reaction mixture in the form of β-sheets. This precipitation hinders cyclization, but it does not hinder the formulation of amino end groups by DMF. Hydrolysis of the *N*-acyl NCA chain end, may then occur during the work-up of the reaction mixture. In the case of poly(d,l-phenylalanine), the higher solubility favors their cyclization.

In Table 5 are depicted few examples of different strategies used by different research groups in order to synthesize cyclic polypeptide structures.

## 6. Non-Linear Polypeptide Architectures: Star, Brush and Highly Branched Polypeptides

### 6.1. Synthesis of Star-Shaped Polypeptides

Two main strategies have been described for the preparation of star-shaped polypeptide using either the core-first strategy and therefore using a multifunctional initiator or using the arm-first strategy that employs a multivalent crosslinking agent to anchor preformed polypeptide chains.

(a) The core-first strategy, also known as divergent approach, uses multifunctional initiators to simultaneously grow the arms required. Many different examples have been reported in the literature by using this strategy that can be classified depending on the nature of the initiator employed, i.e., multifunctional molecules, hyperbranched polymers or dendrimers

**Figure 37:**
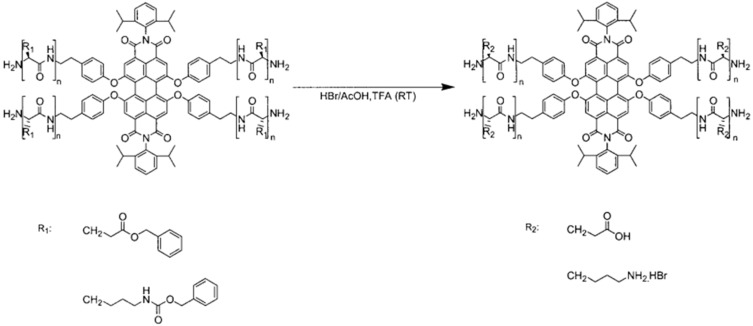
Strategy developed by Klok et al. to fabricate star-shaped fluorescent polypeptides. Reproduced with permission from reference [182].

Perylene derivatives multifunctional molecules with four primary amine groups were synthetized by Klok et al. [182] and employed as initiators for the ROP of BnGlu NCA and Z-Lys NCA (Figure 37). Star-shaped polypeptides with arm lengths ranging from 10 to 200 α-amino acid residues could be readily prepared by variations in the molar ratio of NCA to initiator during the polymerization process. The removal of the side-chain protecting groups afforded unprecedented water-soluble, fluorescent perylene derivatives. These star polypeptides might be of interest for the development of novel fluorescent probes or as traceable, stimuli-sensitive molecular containers. 

Other initiators include cyclotriphosphazenes [183,184], PEO stars [153] or amino terminated multifunctional cyclodextrin [185] and porphyrin/ polyester moieties [186] that have also led to the successful polymerization of NCAs to generate star polypeptides. Inoue et al. [183,184] prepared using this initiators six-arm PBLG polypeptides. In that study, they used hexakis(4-benzylamino-1-oxy)- and hexakis(4-aminophenoxy) cyclotriphosphazene as initiator. Similarly, Karatzas [153]. Karatzas et al., employed a four-arm PEO stars end-functionalized with primary amine groups for the polymerization of BLG-NCA leading to the synthesis of (PEO-*b*-PBLG)_4_ star-block copolymers.

**Figure 38:**
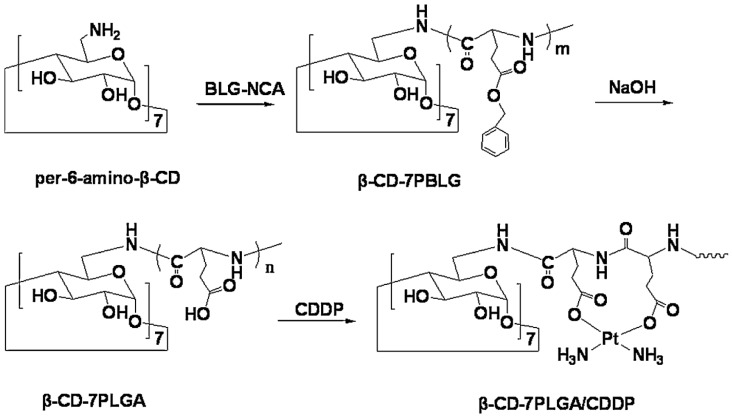
Synthesis scheme of β-CD-7PBLG, β-CD-7PLGA and β-CD-7PLGA/CDDP complex. Reproduced with permission from reference [185].

More recently, the generation of anionic star shaped polypeptides obtained through the ROP of γ-benzyl-l glutamate (BLG) NCAs was reported by Yong et al. [185]. Therefore, β-CD-7PBLG seven-armed polymers were synthesized in DMF by ring-opening polymerization of BLG-NCA initiated by per-6-amino-β-CD (Figure 38). After removal the β-benzyl ester groups of β-CD-7PBLG by hydrolyzation with NaOH, the lyophilized β-CD-7PLGA was easily dissolved in water. The molecular weight of β-CD-7PLGA showed a tendency to increase with the increase of the feed molar ratios of BLG-NCA monomer to amino groups indicating a controlled polymerization although the dispersities reported (1.3–1.5) indicate the formation of relative disperse materials. 

**Figure 39:**
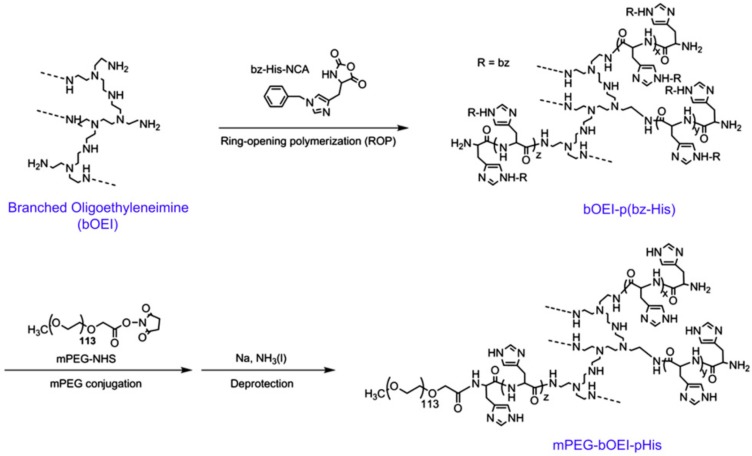
Synthesis of star-shaped block copolymer composed of methoxy poly(ethylene glycol) (mPEG), branched oligoethylenimine (bOEI), and poly(L-histidine) (pHis) via the multi-initiation and ring-opening polymerization (ROP). Reproduced with permission from reference [190].

The second alternative uses hyperbranched polymers as initiators for the ROP of NCAs. These have been mainly obtained using branched polyethyleneimine as multifunctional initiator and PBLG [187,188,189], Histidine [190], Plys [191] as NCA monomers to fabricated star shaped homopolypeptides. For instance, Park et al. [190] reported the synthesis of star-shaped block copolymer composed of methoxy poly(ethylene glycol) (mPEG), branched oligoethylenimine (bOEI), and poly(l-histidine) (pHis) via the multi-initiation and ring-opening polymerization (ROP) of His *N*-carboxy anhydride (NCA) on bOEI with a PEG conjugation. As illustrated in Figure 39, the cyclic histidine monomer bzHis-NCA was first prepared and polymerized using six primary amine groups of bOEI. The resulting bOEI-p(bzHis) was confirmed by ^1^H NMR. Although some of the bOEI primary amines were spent to initiate the ROP of bzHisNCA, an equal number of primary amines was generated at the ends of p(bzHis) in bOEI-p(bzHis). The newly formed primary amines allowed for a coupling reaction with activated mPEG-COOH, resulting in the synthesis of mPEG-bOEI-p(bzHis). The final product, mPEG-bOEI-pHis (POH), was prepared by deprotection of mPEG-bOEI-p(bzHis) and was observed to be highly water-soluble. 

**Figure 40:**
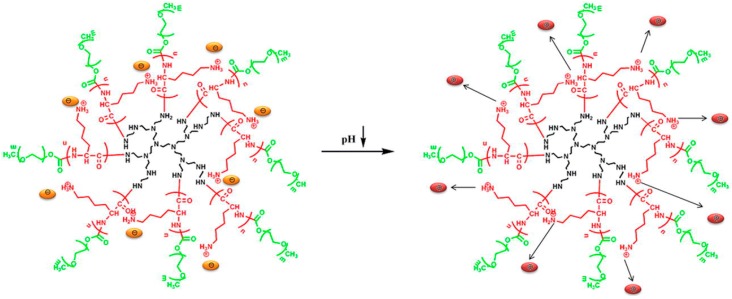
Chemical structure of the hydrophilic star block co-polymers and their use as protein nanocarrier developed by Yan et al. Reproduced with permission from reference [192].

Hydrophilic star block co-polymers were synthesized by Yan et al. [192] using PLL as amino acid and by Huang et al. [193] using PLGA. In the work of Yan et al. they described the preparation, the characterization as well as the evaluation as protein nanocarrier [192]. The star block co-polymer was composed of a hyperbranched polyethylenimine (PEI) core, a poly(Llysine) (PLL) inner shell, and a poly(ethylene glycol) (PEG) outer shell (Figure 40). The model protein selected was insulin that could be rapidly and efficiently encapsulated by the synthesized polymer in aqueous phosphate buffer at physiological pH. Complexation between PEI-PLL-*b*-PEG and insulin was investigated using native polyacrylamide gel electrophoresis. The encapsulated insulin demonstrated sustained release at physiological pH and showed accelerated release when the pH was decreased. The insulin released from the star block co-polymer retained its chemical integrity and immunogenicity.

**Figure 41:**
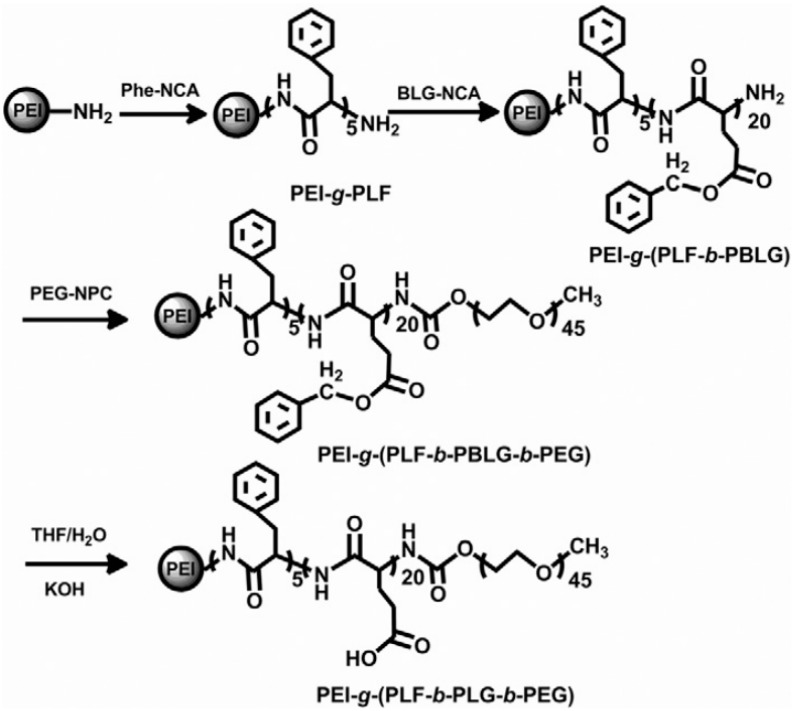
Synthetic route of the star-block quadripolymer, PEI-*g*-(PLF-*b*-PLG-*b*-PEG). The synthesis of only one arm is shown. Reproduced with permission from reference [194].

Finally, the synthesis of polypeptide-based star-block quadripolymers and its use as unimolecular nanocarriers for the simultaneous encapsulation of hydrophobic and hydrophilic guests was reported by Li et al. [194]. The star-block quadripolymers PEI-*g*-(PLF-*b*-PLL-*b*-PEG) and PEI-*g*-(PLF-*b*-PLG-*b*-PEG) comprise a polyethylenimine (PEI) core, an amphiphilic copolypeptide poly(l-phenylalanine)-*b*-poly(l-lysine) (PLF-*b*-PLL) or poly(l-phenylalanine)-*b*-poly(l-glutamic acid) (PLF-*b*-PLG) inner shell, and a poly(ethylene glycol) (PEG) outer shell (Figure 41). The star-block quadripolymers were obtained by sequential ring-opening polymerizations of l-phenylalanine *N*-carboxyanhydride and *N*^ε^-benzyloxycarbonyl-l-lysine *N*-carboxyanhydride or γ-benzyl-l-glutamate *N*-carboxyanhydride initiated by the terminal primary amines of PEI. Subsequently, the periphery was PEGylated, and the poly(l-lysine) or poly(l-glutamic acid) side chains were deprotected. These polymers were well dispersed in aqueous solutions and resembled amphiphilic unimolecular micelles were able to solubilize nonpolar model compounds through hydrophobic interactions. Moreover, these polymers could efficiently encapsulate hydrophilic model compounds via electrostatic interactions or even entrap hydrophobic and hydrophilic model compounds in the site-isolated state simultaneously. The entrapped hydrophilic model compounds demonstrated sustained release at physiological pH and accelerated release when the pH was either increased or decreased. The simultaneous encapsulation of versatile guest molecules as well as the pH-responsive releasing properties of these star-block quadripolymers could be potentially useful in the controlled drug co-delivery applications.

Finally, the third alternative to prepare star shaped polypeptides by the core-first approach involves the use of dendrimers functionalized with a precise number of primary amine groups as multifunctional initiators for the polymerization of NCAs. In fact, as the dendrimer generation increases, the peripheral amine functionality also increases permitting the synthesis of different polypeptide star polymers. Polypropylene imine (PPI) and poly(amidoamine) (PAMAM) dendrimers are the two most widely employed dendrimers in NCA polymerization partly owing to their commercial availability. The first case of polypeptide growth from a dendritic core via NCA polymerization was reported by Okada [195], who constructed a short armed glycosylated star polypeptide from the ROP of glycosylated NCA via generation 3 PAMAM. The polymers, dubbed “sugar balls” were shown to be well-defined in terms of molecular weight distribution with NCA polymerisation proceeding via NAM with all the amino peripheral groups employed for the polymerization. Other example, Harada et al. [196,197] synthesized a polyamidoamine dendron (PAMAM) carrying Boc-protected amine groups that upon removal were employed as initiators for the polymerization of ZLL-NCA. 

**Figure 42:**
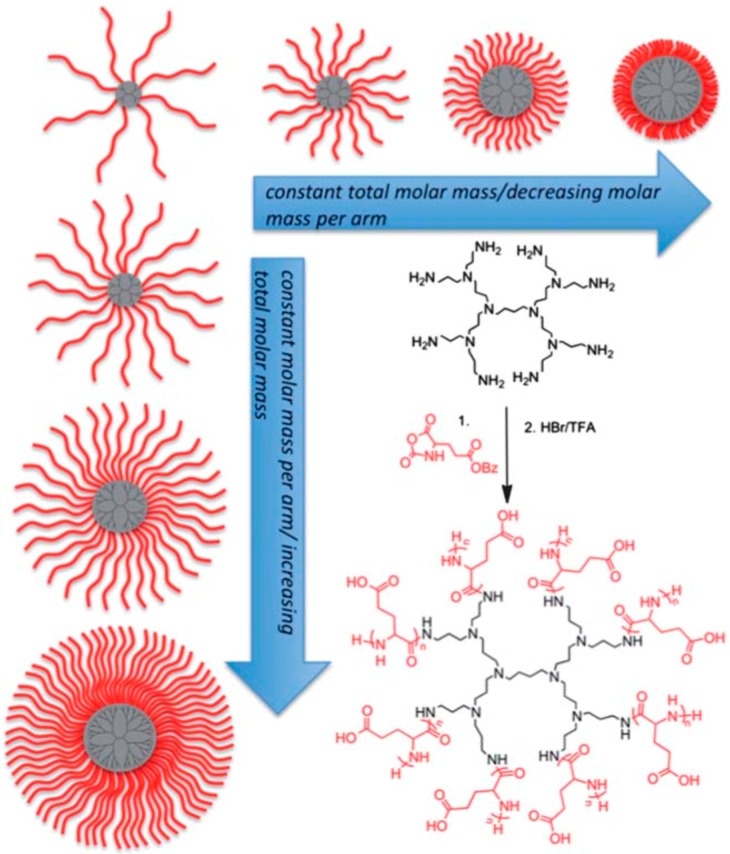
Diversity of synthesized star polypeptides and reaction scheme for the synthesis of poly(l-glutamic acid) star polypeptides using polypropylene imine (PPI) dendrimers as initiators. Reproduced with permission from reference [199].

However, probably one of the most relevant studies was reported by Byrne et al. using different generations of polypropylene imine (PPI) dendrimers as initiators [198,199]. A series of well-defined star-shaped polypeptides were successfully synthesized by the ring opening polymerisation (ROP) of the *N*-carboxyanhydride (NCA) of *N*^ε^-carbobenzyloxy-l-lysine (ZLL) using a range of generations of polypropylene imine (PPI) dendrimers as multifunctional initiators (Figure 42). The monomer feed ratio and dendrimer generation were varied to afford a series of polypeptide dendrimer hybrids with superior structural versatility and functionality. Subsequent protecting group removal yielded star-shaped poly(l-lysine)s with variable polypeptide chain length and arm multiplicity. The same approach was later extended to the fabrication of star-shaped architectures with a maximum of 8 to 64 poly(γ-benzyl-l-glutamate) (PBLG) arms. By deprotection, the PBLG star polypeptides were converted into poly(l-glutamic acid) (PGA) star polypeptides. In a very recent work these PGA based star polypeptides were glycosylated. Glycosylation of star-shaped poly(glutamic acid) resulted in the formation of a diverse range of glycopolypeptide architectures with tuneable degree of sugar conjugation [200]. The secondary structure of the branched glycopolypeptides was studied as a function of the degree glycosylation. The bioactivity of the described glycopoly-peptides toward the lectin ConA was investigated and was shown to be architecture dependent.

(b) The arm-first strategy uses a multivalent crosslinking agent where the polypeptide chains are covalently anchored. This strategy presents several important advantages over the core-first strategy including the facility for the characterization of arms and final star product, i.e., arm length and star polymer functionality can be readily and accurately determined. However, this methodology presents several drawbacks including the extended linking reaction time and the need to remove unreacted linear arms often using the crude purification process of fractionation. 

**Figure 43:**
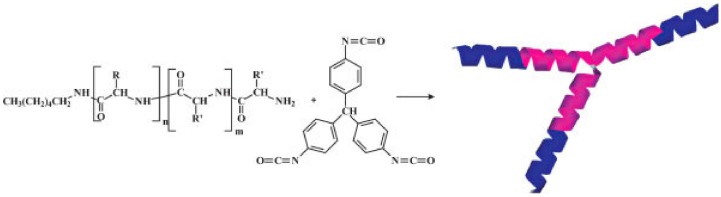
Strategy reported by Aliferis et al. used to fabricate three-arm polypeptides by the arm-first strategy. Reproduced with permission from reference [201].

This strategy was employed by Aliferis et al. [201] for the synthesis of, both, star homopolypeptides based on (PBLG)_3_ and (PZLL)_3_ as well as 3-arm star-block copolypeptides, PZLL-*b*-PBLG_3_ based on a combination of both amino acids. The strategy employed is based on the use of three-valent isocyanate crosslinking agent that readily reacted with the amino end-terminal group of the polypeptide (Figure 43). In order to efficiently link the polypeptide chains, the authors employed an excess of the living arms and carried out the salting-out technique to purify and obtain the pure product. 

**Figure 44:**
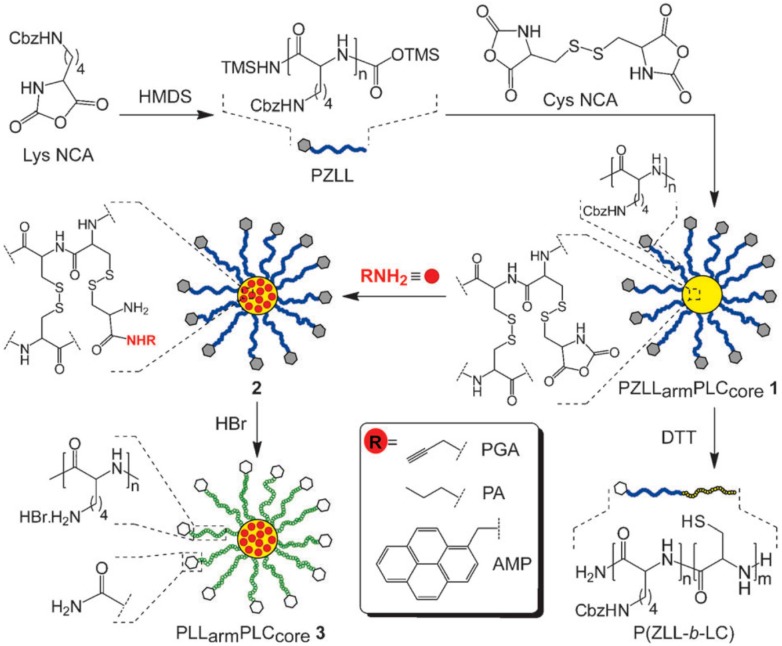
Synthesis of amino acid-based Core Cross-linked Star (CCS) polymers having hierarchical functionalities via a one-pot arm-first strategy. Reproduced with permission from reference [202].

Alternative strategies to fabricate star polypeptides with a larger number of arms have been reported. In this context, Qiao group investigated extensively this method for the preparation of star shaped polypeptides by the preparation of Core Cross linked Star (CCS) polymers. [202,203,204] Their approach to prepare multiply functionalized CCS polymers comprised entirely of amino acids via ring opening polymerization (ROP) of amino acid *N*-carboxyanhydrides (NCA) in a one-pot, arm-first approach (Figure 44). The CCS polymers had selectively degradable cores and possessed hierarchical functionalities spanning from the peripheral groups, along the arms, as well as on the core itself. ROP of *N*^ε^-Z-l-lysine NCA initiated with the secondary amine, hexamethyldisilazane (HMDS), afforded linear living poly(*N*^ε^-Z-l-lysine) (PZLL)_7_ which served as the ‘arm’ or macroinitiator (MI) for star formation. Subsequent addition of l-cystine NCA, which acts as a cross-linker, resulted in the formation of poly(*N*^ε^-Z-l-lysine)arm poly(l-cystine)core (PZLL-arm-PLC-core) CCS polymer 1. Unreacted pendant NCAs within the core of the star 1 allowed for facile post-functionalization through reaction with primary amines bearing different functional groups, such as propargylamine (PGA), propylamine (PA) and aminomethyl pyrene (AMP) to yield core-functionalized stars 2. Deprotection of the carboxybenzyl (Cbz) protecting groups from the side-chains of the PZLL arms of star 2 gave the water soluble poly(L-lysine)arm poly(l-cystine)core (PLL-arm-PLC-core) CCS polymer 3. The degradability of the CCS polymers was demonstrated by cleavage of the disulfide bridge in the core with reducing agents such as dithiothreitol (DTT), which yielded the star’s linear poly((*N*^ε^-Z-l-lysine)-*b*-(l-cystine)) (P(ZLL-*b*-LC)) block copolymer constituents.

Other groups have also prepared CCS star polymers with, for instance, polypeptide periphery and styrene cores synthesized via the cross-linking of styrenic terminated PBLG arms with divinylbenzene (Heise et al. [205,206]) or alkyne terminated PBLG and multifunctional azide terminated polyhedral oligomeric silsesquioxane (POSS) [207].

**Figure 45:**
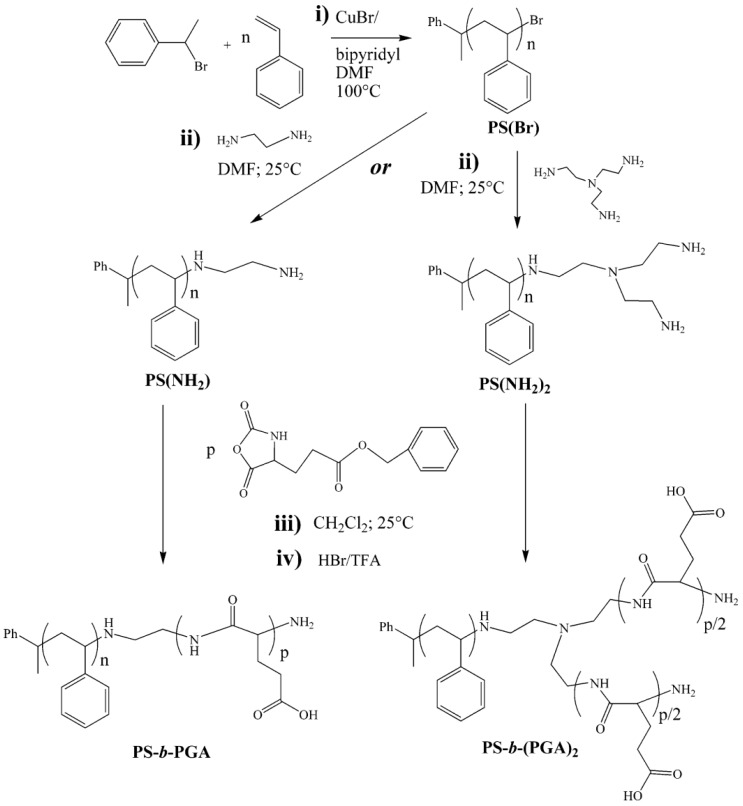
Synthetic strategy for miktoarm star hybrid-copolypeptides reported by Babin et al. Reproduced with permission from reference [11].

(c) Combined strategies to fabricate star-shaped polypeptides. Hybrid star-shaped polypeptide structures have been also prepared by combining other living/controlled polymerization methodologies with ROP of polypeptides. An illustrative example of this combination was reported by Babin et al. [11,208]. Babin and coworkers combined ATRP and ROP for the synthesis of PS(PBLG)_2_ miktoarm star copolymers, as depicted in Figure 45. The four-step synthetic strategy combined ATRP of styrene, selective derivatization of PS chain-ends, ROP of γ-benzyl-l-glutamate *N*-carboxyanhydride, and a final treatment under acidic conditions in order to remove the γ-benzyl group rendering from the amphiphilic miktoarm.

**Figure 46:**
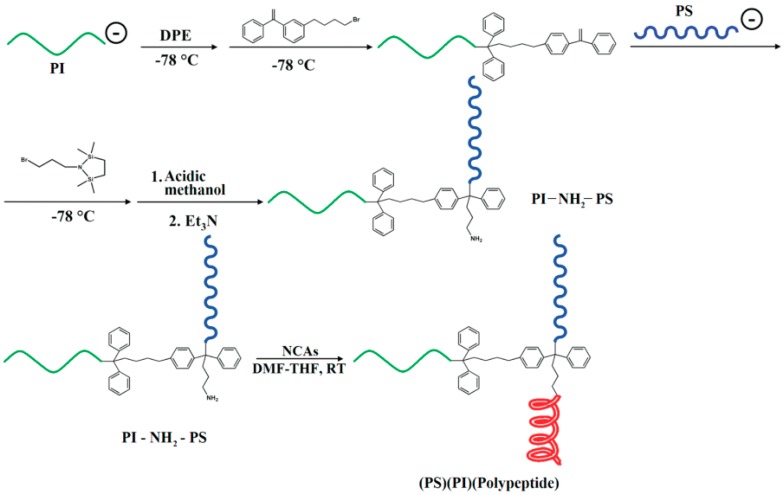
Synthesis of the 3-Miktoarm Star *Chimeras* (PS)(PI)(Polypeptide). Reproduced with permission from reference [177].

Other miktoarm structures have equally being reported by other groups. For instance, Cho et al. [209] used a PEO bearing two central- and two end-amine groups as a macroinitiator for the polymerization of BLG-NCA, leading to the synthesis of (PEO-*b*-PBLG)_2_(PBLG)_2_ miktoarm star copolymers. Also, Sun et al. [210] prepared similar structures using, in this case, a trifunctional initiator, i.e., 2-benzyloxycarbonylamino-1,3-propanediol. Finally, more intricate structures were synthesized by Karatzas et al. [177] combining anionic polymerization and ROP of NCAs. They employed high vacuum conditions in order to avoid any contamination, necessary for the preparation of a variety of well-defined miktoarm star hybrids. An example of these complex structures is depicted in Figure 46. In this case, polystyrene-*b*-polyisoprene functionalized with one NH_2_ group at the junction (PS-NH_2_-PI) was synthesized by the reaction of PI-D with polystyryllithium, followed by the reaction with acidic methanol and their corresponding deprotection. The amino group was then employed to initiate the ROP NCA.

A similar strategy was recently employed by Zhao et al. [74]. They reported that based on hydrogen-bonding organocatalysis, living ring-opening polymerization of *N*-carboxyanhydride of α-amino acids using aminoalcohols as initiators in the presence of *N*,*N*′-bis[3,5-bis(trifluoromethyl)phenyl]thiourea (TU-S) was achieved. The thiourea provided, through hydrogen bonding, simultaneous activation of NCA monomers/reversible deactivation of polymer chain-ends/silencing of the tertiary amine and thus allowed the polymerization to proceed in a highly controllable mode. For example, by using *N*,*N*-dimethyl ethanolamine (DMEA), as an initiator in the presence of TU-S, a series of well-defined linear polypeptides with differently designed M_ns_ (3.01 × 10^4^–18.10 × 10^4^) and low PDI values (1.02–1.05) were successfully synthesized. This general strategy was also extended to the synthesis of well-defined di- and multi-armed polypeptides by using di-, tri-, or tetra-aminoalcohol initiators (methyldiethanolamine (MDEA), triethanolamine (TEA) or *N*,*N*,*N*′,*N*′-tetrakis(2-hydroxyethyl)ethylenediamine (THEED)) in the presence of TU-S. 

### 6.2. Grafted Polypeptides and Other Highly Branched Structures

#### 6.2.1. Methods for the Preparation of Polypeptide Brushes

In addition to the non-linear star-shaped polypeptides, other grafted and highly branched polypeptide structures have been also reported. For the preparation of branched grafted polypeptides, the traditionally reported strategies “grafting onto” [211,212,213,214,215], “grafting from” [216,217,218] and “grafting through” [219,220] methodology have been employed. 

Grafting onto Methodologies

**Figure 47:**
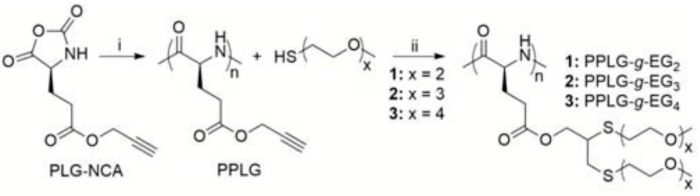
The synthetic route to PPLG-*g*-EGx by a combination of ROP of PLG-NCA and thiol-yne photochemistry. Reagents and conditions: (i) HMDS, THF/DMF 1:1 *v*/*v*, rt, 48 h. (ii) DMPA, DMF, hν, 3 h. Reproduced with permission from reference [215].

Fu et al. [215] obtained poly(l-glutamate)-*g*-oligo(ethylene glycol) graft polymers by a combination of NCA polymerization of γ-propargyl-l-glutamate NCA and thiol-yne photoaddition using thiol-terminated oligo(ethylene glycol). As depicted in Figure 47, a series of poly(l-glutamate) bearing Y-shaped oligo(ethylene glycol)x (OEGx) side-chains (PPLG-g-EGx, x = 2, 3 and 4) were synthesized via a combination of ring opening polymerization (ROP) of γ-propargyl-l-glutamate *N*-carboxyanhydride (PLG-NCA) with thiol-yne photoaddition. Interestingly, PPLG-*g*-EG3 and PPLG-*g*-EG4 were soluble in water and displayed fully reversible thermal-responsive behaviors. Additionally, the polypeptides showed redox-responsive properties along with the conformation associated water solubility due to the presence of thioether groups in side-chains.

Grafting from Methodologies

**Figure 48:**
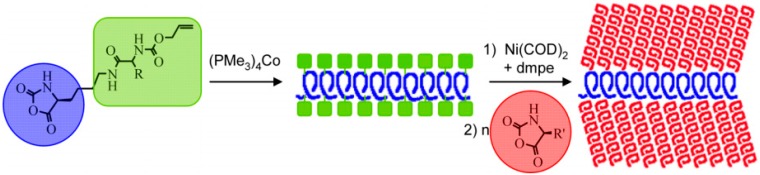
One-pot synthesis of cylindrical brush copolypeptides reported by Deming et al. The *N*-carboxyanhydride (NCA) component (blue) of *N*^ε^-(alloc-l-methionyl)-l-lysine NCA was first polymerized using (PMe_3_)_4_Co initiator to give a linear polypeptide that bears pendant initiator precursors (green). Side-chain initiators were then activated by using dmpeNi(COD), followed by addition of a second NCA monomer (red) to give the brush copolymers. Reproduced with permission from reference [218].

An illustrative example of the preparation of polypeptide brushes was reported by Deming et al. [218]. They described a methodology for the synthesis of cylindrical copolypeptide brushes via *N*-carboxyanhydride (NCA) polymerization using a tandem catalysis approach that allowed preparation of brushes with controlled segment lengths, in a single-step procedure requiring no intermediate isolation or purification stages. As shown in Figure 48, to obtain high-density brush copolypeptides, they used a “grafting from” approach where alloc-α-aminoamide groups were installed onto the side chains of NCAs to serve as masked initiators. These groups were inert during cobalt-initiated NCA polymerization and gave allyloxycarbonyl-α-aminoamide-substituted polypeptide main chains. The alloc-α-aminoamide groups were then activated in-situ using nickel to generate initiators for the growth of side-chain brush segments. The use of stepwise tandem cobalt and nickel catalysis was found to be an efficient method for preparation of high-chain-density, cylindrical copolypeptide brushes.

**Figure 49:**
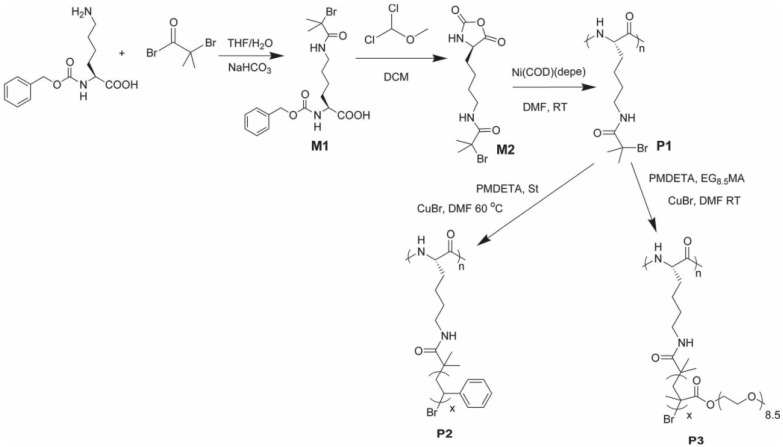
Synthetic route to poly(Br-l-lysine) (PBrLL) (**P1**), PLL-*g*-PS (**P2**), and PLL-*g*-PEGMA (**P3**) polypeptide bottlebrushes. Reproduced with permission from reference [52].

Molecular bottlebrushes based on poly-l-lysine (PLL) as backbone were reported by Liu et al. [52] via ROP and subsequently ATRP (Figure 49). The approach started with the preparation of *N*^ε^-bromoisobutyryl functionalized N^α^-CBZ-l-lysine and converted in polymerizable α-amino acid *N*-carboxyanhydride (NCA). Then, the NCA was polymerized using Ni(0) transition metal complex to give well-defined bromo-functionalized homopolypeptide (PBrLL), from which the authors prepared two types of polypeptide bottlebrushes with polystyrene and poly(oligoethylene glycol methacrylate) as side-chains. PBrLL macroinitiator was demonstrated to have high initiation efficiency for ATRP, which allowed good control over side-chain length. 

Polyglutamic acid with chloride in the side chain was also used in a “grafting from” approach by the group of Chen [221,222]. Starting from α-helical poly(2-chloro ethyl glutamate), atom-transfer radical polymerization (ATRP) was used to graft methoxy di(ethylenoxid) methacrylate from the side chain. With this strategy Chen et al. [221], synthesized a novel thermo-responsive polypeptide, poly(l-glutamate)-*g*-poly(2-(2-methoxyethoxy)ethyl methacrylate) (PLG-*g*-PMEO2MA) was prepared by the combination of ROP of γ-2-chloroethyl-l-glutamate-*N*-carboxyanhydride (CELG-NCA) using *n*-hexylamine as the initiator and subsequent ATRP of 2-(2-methoxyethoxy)-ethyl methacrylate (MEO_2_MA) monomer. This model thermo-responsive graft copolymer revealed the high efficiency of “grafting from” polymer side chains while maintaining the a-helical polypeptide backbone.

Grafting through Methodologies

**Figure 50:**
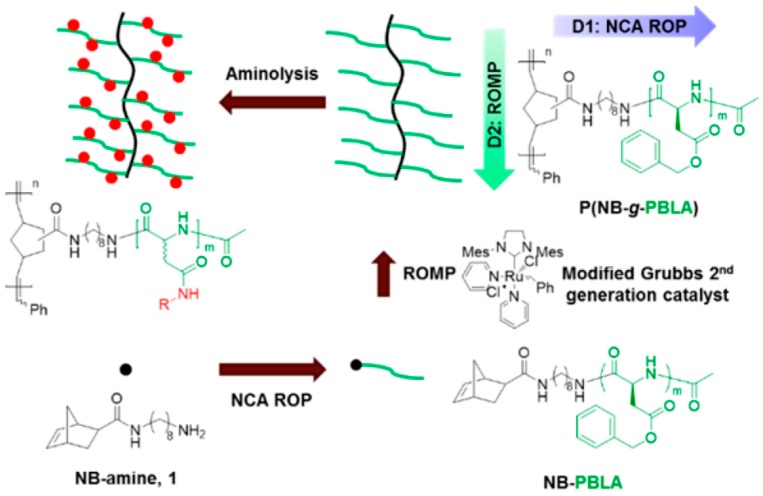
Synthetic design of polypeptide molecular brushes via “grafting through” strategy with postpolymerization modification using aminolysis of PBLA brush side chains. Reproduced with permission from reference [220].

An illustrative example of the use of this strategy was reported Fan et al. [220] for the preparation of poly(norbornene-*graft*-poly(β-benzyl-l-aspartate)) (P(NB-*g*-PBLA)). They integrated both *N*-carboxyanhydride ring-opening polymerizations (NCA ROPs) and ring-opening metathesis polymerizations (ROMPs) and were able to independently construct in controlled manners both the desirable segment lengths of polypeptide side chains as well as the polynorbornene brush backbones (Figure 50). The N_2_ flow accelerated NCA ROP was utilized to prepare polypeptide macromonomers with different lengths initiated from a norbornene-based primary amine, and those macromonomers were then polymerized via ROMP. In order to increase the graft density a mixture of dichloromethane and an ionic liquid was required as the solvent system. This solvent mixture allowed the construction of molecular brush polymers having densely-grafted peptide chains emanating from a polynorbornene backbone, poly(norbornene-*graft*-poly(β-benzyl-l-aspartate)) (P(NB-*g*-PBLA)). Highly efficient postpolymerization modification was achieved by aminolysis of PBLA side chains to obtain functional moieties onto the molecular brushes.

#### 6.2.2. Dendritic Graft, Arborescent and Hyperbranched Polypeptide Architectures

**Figure 51:**
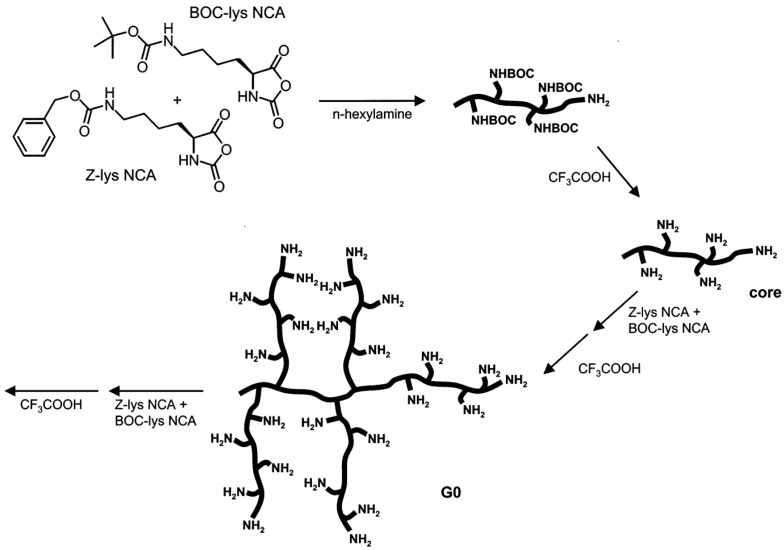
Strategy reported by Klok and Rodriguez-Hernandez for the fabrication of dendritic-graft polypeptides. Reproduced with permission from reference [223].

The synthesis of the dendritic-graft polypeptides was described by Klok and Rodriguez-Hernandez [223] and is outlined in Figure 51. The synthetic strategy was based on the ring-opening copolymerization (ROCP) of two orthogonally *N*^ε^-protected l-lysine NCAs. One of the monomers contained a temporary protective group, which could be removed under relatively mild conditions. The ε-NH_2_ group of the other l-lysine monomer was masked with a permanent protective group, stable under the conditions applied for the removal of the temporary protective group. The permanent protective group was removed in the very last step of the synthesis. In the first synthetic step, a primary amine was used to initiate the ROCP of the two NCAs to prepare the core of the targeted polypeptide. Removal of the temporary protective group generated a number of primary amine groups, which can act as an initiator to graft the first generation of peptide arms onto the core. Repetition of this NCA ring-opening polymerization/deprotection cycle yielded highly branched, or dendritic-graft, polypeptides.

**Figure 52:**
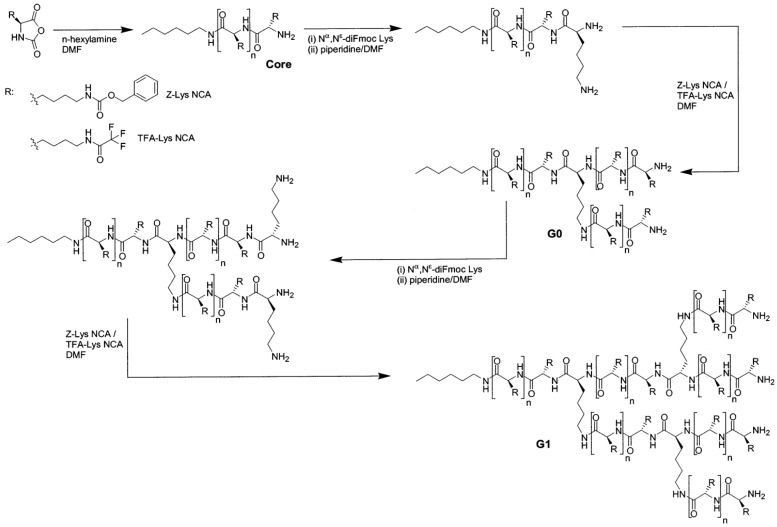
Synthetic strategy for the fabrication of highly branched poly(l-lysine). Reproduced with permission from reference [224].

Highly branched poly(l-lysine) was also prepared by a repetitive sequence of NCA polymerization and end-functionalization/deprotection reactions as depicted in Figure 52 [224]. Z-Lys-NCA or *N*^ε^-trifluoroacetyl-l-lysine-NCA was initially polymerized with *n*-hexylamine. The polymer was then end-functionalized by reaction with *N*^α^,*N*^ε^-diFmoc-l-Lys (Fmoc: 9-fluorenylmethoxycarbonyl) under standard peptide coupling conditions. Deprotection of the *N*^α^,*N*^ε^-diFmoc Lys end group produced two new primary amine groups that could initiate the polymerization of the second generation of branches. Repetition of this ring-opening polymerization-end functionalization sequence afforded highly branched poly(*N*^ε^-benzyloxycarbonyl-l-lysine) (poly(Z-Lys)) and poly (*N*^ε^-trifluoroacetyl-l-lysine) (poly(TFA-Lys)) in a small number of straightforward synthetic steps. Removal of the side-chain protective groups yielded water-soluble and highly branched poly(l-lysine)s.

**Figure 53:**
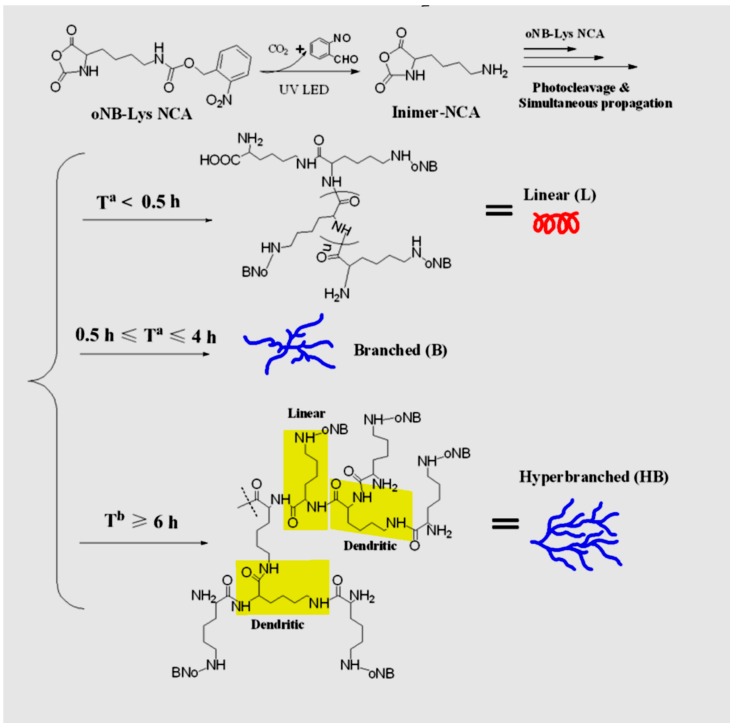
Illustration for UV-Triggered ROP of oNB-LysNCA in One Pot and at Room Temperature. ^a^ Polymerize in dark place after initial UV-irradiation. ^b^ Consecutive irradiation until complete monomer conversion. Reproduced with permission from reference [225].

Very recently, Li and Dong [225] reported the use of phototriggered ring-opening polymerization of a photocaged l-Lysine *N*-Carboxyanhydride to synthesize hyperbranched and linear polypeptides. In their strategy, they combined the inimer (initiator + monomer) ring-opening polymerization (ROP) and photocaged chemistry to prepare hyperbranched and linear polypeptides without addition of any initiator/catalyst. The approach, schematically shown in Figure 53, used a photocaged *N*^ε^-(*o*-nitrobenzyloxycarbonyl)-l-lysine-*N*-carboxyanhydride (oNB-LysNCA) that possessed intrinsic photosensitivity and could be straightforwardly transformed into an activated AB* inimer-type α-amino acid *N*-carboxyanhydride (NCA) upon UV-LED exposure. As a result, the activated inimer contains a primary ε-amine, which further triggered ROP to produce linear and/or hyperbranched polypeptides in one pot and at room temperature. More interestingly, as the authors reported by tuning the UV irradiation time or intensity, this methodology permitted the synthesis of either linear polypeptide with a high M_w_ or (hyper)branched polypeptides with tunable M_w_ (1.4–73.5 kDa) and degree of branching (0.09–0.60).

## 7. Summary and Conclusions

The ROP NCAs is today an interesting synthetic tool for the synthesis of functional polypeptides with variable chemical composition and topology while permitting the fabrication of high molecular weight polypeptides with narrow polydispersities. In this review, we provided a general overview of this type of polymerization including the preparation of functional monomers, the mechanisms and the recent developments in the ROP NCAs that permit a better control over dispersity and chain length. These improvements are, without any doubt, the consequence of a large amount of work reported focused on the fabrication of multiple macromolecular architectures and the large variety of applications explored for these materials. Block copolypeptides able to form nanocapsules or highly branched polypeptides have been extensively explored for the delivery of therapeutics including drug, proteins or even genes. Functional polypeptides are at the base for the fabrication of many different types of hydrogels that have been and are currently being evaluated for tissue engineering purposes. Finally, another interesting example is the use of hybrid polypeptide materials for the fabrication of tissue engineering scaffolds since polypeptides such as poly(l-lysine) promote cell adhesion and proliferation.

All these applications require a thorough macromolecular design. However, with a large number of different synthetic strategies at hand the precise fabrication of a particular polypeptide structure can be realized and tailored in order to meet the requirements of a particular biomedical or pharmaceutical need.

## Figures and Tables

**Table 1:** Illustrative examples of functional NCAs. Adapted from reference [13].

**Alkyne-Azide [2 + 3] Huisgen Cycloaddition**			
PG-NCA [33,34,35,36] 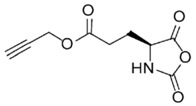		PG-Gly-NCA [37,38] 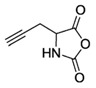	
CP-NCA [39] 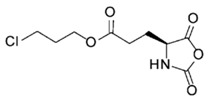		Anl-NCA and Anv-NCA [40] 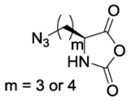	
**Thiol-Ene Reactions**			
DL-Allylglycine [41,42] 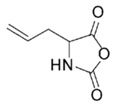	AL-NCA [43] 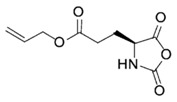		AOB-Glu-NCA [44] 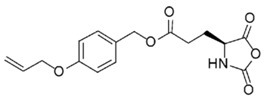
**Mimics of Glycosylated Peptides and Proteins**			
α-Glyco-Lys-NCAs [45,46,47] 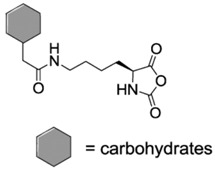		Glyco NCAs (R = Ac4Gal, Ac4Glu or Ac7Lac; X = O or S; R0 = Me or H) 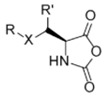	
**Thermo-Responsive Polypeptides**			
L-EGxGlu-NCA [48] 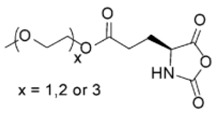		EGxMA-Cys-NCA (R = CH3) and EGxA-Cys-NCA (R = H) [49] 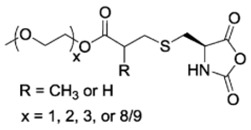	
**Photo-Responsive**			
NBC-NCA [50] 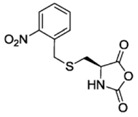		DMNB-Glu-NCA (R = OMe) [51] 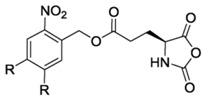	
**ATRP to Prepare Molecular Bottlebrush**		**Organic Semiconductor Unit; Organic Photovoltaic and Organic Field Effect Transistor**	
Br-Lys-NCA [52] 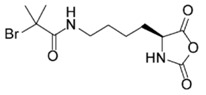		Hexithiophene-LysNCA [53] 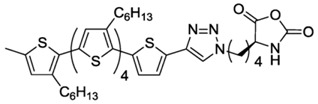	

**Table 2:** Characterization of poly(*N*^ε^-trifluoroacetyl-l-lysine) samples by nonaqueous capillary electrophoresis (NACE). Table reproduced with permission from reference [78].

M/I	Temperature (°C)	Living Polymers (°C) ^a^	Dead Polymers (Peaks B and C) (%) ^a^
50	50	20	80
50	Room	22	78
50	0	99	1

^a^ From NACE experiments.

**Table 3:** Illustrative examples of block copolypeptides reported in the literature.

Type of Block Copolymer	Block Copolypeptide	Reference
Diblock	poly[**γ**-benzyl-l-glutamate-*b*-(l-glutamic Acid)]	[121]
Diblock	*N*^ε^-4-phenylbenzamido-l-lysine-*b*-*N*^ε^-trifluoroacetyl-l-lysine	[120]
Diblock	*o*-(tetra-*o*-acetyl-d-glucopyranosyl)-l-serine-NCA-*b*-alanine NCA	[119]
Diblock	poly(γ-benzyl-l-glutamate)-*b*-polyglycine	[122]
Diblock	poly[**γ**-benzyl-l-glutamate-*b*-(poly(*N*^ε^-carbobenzoxy-l-lysine)], poly[**γ**-benzyl-l-glutamate-*b*-polyglycine], poly[**γ**-benzyl-l-glutamate-*b*-poly(l-tyrosine)], poly[**γ**-benzyl-l-glutamate-*b*-poly(poly(l-leucine)].	[76]
Diblock	poly(γ-benzyl-l-glutamate)-*b*-poly(l-lysine)	[123]
Diblock	poly[**γ**-benzyl-l-glutamate-*b*-(poly(*N*^ε^-carbobenzoxy-l-lysine)], and block copolypeptides containing l-leucine and l-proline)	[130]
Diblock	poly(*N*^ε^-2[2-(2-methoxyethoxy)ethoxy]acetyl-l-lysine)-*b*-poly(l-aspartic acid, sodium salt)	[131]
Diblock	poly(l-lysine Hbr) or poly(l-glutamic acid sodium salt), and helical, hydrophobic segments of poly(l-leucine)	[132,133]
Diblock	diblock copolypeptides of hydrophilic l-lysine or l-glutamic acid and hydrophobic leucine or valine	[134]
Diblock	poly(l-lysine)-*b*-poly(l-glycine), poly(l-lysine)-*b*-poly(l-alanine), poly(l-lysine)-*b*-poly(l-phenylalanine)	[135]
Diblock	poly(*S*-(*o*-nitrobenzyl)-l-cysteine)-*b*-poly(ethylene glycol) (PNBC-*b*-PEO) block copolymers	[50]
Diblock	Hydrophilic (glutamic acid or lysine) and one nonpolar block (alanine) or with both hydrophilic blocks with opposite charges (glutamic acid and lysine)	[136]
Diblock	Poly(l-lysine)-*b*-poly(l-glycine)	[137,138]
Diblock	poly(l-lysine)-*b*-poly(l-phenylalanine)	[139]
Diblock	poly(l-methionine)_65_-*b*-poly(l-leucine_0.5_-stat-l-phenylalanine_0.5_)_20_	[140]
Diblock/Triblock	poly-l-lysine-*b*-poly-l-leucine and poly-l-lysine-*b*-poly-l-leucine-*b*-poly-l-lysine	[128]
Triblock	poly[(**γ**-benzyl-l-glutamate)-*b*-(l-leucine)-*b*-(**γ**-benzyl-l-glutamate)] and the corresponding poly[(l-Glutamic Acid)-*b*-(l-leucine)-*b*-(l-Glutamic Acid)], poly(*N*^ε^-carbobenzoxy-l-lysine)-*b*-(**γ**-benzyl-l-glutamate)-*b*-(poly-*N*^ε^-carbobenzoxy-l-lysine)	[76,126,127,141]
Triblock	poly(l-homoarginine HCl)m-block-poly(Lglutamate Na)n-block-poly(l-leucine)20	[6]
Triblock	poly[(*N*^ε^-carbobenzoxy-l-lysine)-*b*-poly(l-leucine)-*b*-poly(*N*^ε^-carbobenzoxy l-lysine)]	[128,129]
Tetrablock	Poly(γ-benzyl-l-glutamate)-*b*-poly(l-alanine)-*b*-poly(*N*^ε^-benzyloxycarbonyl-l-lysine)-*b*-poly(β-benzyl-l-aspartate)	[79]

**Table 4:** Illustrative list of examples of hybrid block copolypeptides reported in the literature.

Type of Block Copolymer (Diblock, Triblock, Pentablock…)	Non-Polypeptidic Block	Hybrid Copolymer Prepared	References
	Polyethylene oxide	polyethyelene oxide-*b*-poly(γ-benzyl-l-glutamate)	[164,165]
	Polyethylene oxide	polyethyelene oxide-*b*-poly(-benzyl-l-aspartate)	[165]
	Polyethylene oxide	polyethyelene oxide-*b*--*b*-poly(l-valine/l-leucine)	[166]
	Polypseudorotaxane	poly(γ-benzyl-l-glutamate)-*b*--*b*-polypseudorotaxane	[7]
Diblock	poly(ε-caprolactone)	poly(ε-caprolactone)-*b*-poly(γ-benzyl-l-glutamate)	[167]
Diblock	Polylactic acid	polylactic acid-*b*-poly(l-Alanine), polylactic acid-*b*-poly(l-phenylalanine), polylactic acid-*b*-poly(l-leucine), polylactic acid-*b*-poly(γ-benzyl-l-glutamate) and polylactic acid-*b*-Poly(benzyl-l-aspartate)	[10,157,158]
Diblock	Poly(ε-caprolactone)	poly(ε-caprolactone)-*b*-poly(γ-benzyl-l-glutamate)	[156]
Diblock	Poly(ε-caprolactone)	poly(ε-caprolactone)-*b*-poly(l-Glycine), poly(ε-caprolactone)-*b*-poly(l-alanine), poly(ε-caprolactone)-*b*-poly(l-Phenylalanine), poly(ε-caprolactone)-*b*-poly(γ-benzyl-l-glutamate)	[168]
Diblock	Poly(*N*-isopropylacrylamide)-	poly(*N*-isopropylacrylamide)-*b*-poly(γ-benzyl-l-glutamate)	[145,151]
Diblock	Poly(2,7-dibromo-9,9-dihexylfluorene)	2,7-dibromo-9,9-dihexylfluorene-*b*-poly(γ-benzyl-l-glutamate)	[161]
Diblock	Poly(*N*-Vinylpirrolidone)	poly(*N*-Vinylpirrolidone)-*b*-poly(γ-benzyl-l-glutamate), poly(*N*-Vinylpirrolidone)-*b*--*b*-poly(Z-l-Lysine) and poly(*N*-Vinylpirrolidone)-*b*-poly(γ-benzyl-l-glutamate)-*b*-poly(Z-l-Lysine)	[153]
Diblock	Polysaccharide	polysaccharide-*b*-poly(γ-benzyl-l-glutamate)	[162]
Diblock	Poly(ferrocenyldimethylsilane)	poly(ferrocenyldimethylsilane)-*b*-poly(γ-benzyl-l-glutamate)	[169]
Diblock	Poly(1-(3-chloropropyl)-2,2,5,5-tetramethyl-1-aza-2,5-disilacyclopentane)	1-(3-chloropropyl)-2,2,5,5-tetramethyl-1-aza-2,5-disilacyclopentane-*b*-poly(γ-benzyl-l-glutamate)	[159]
Diblock	Poly(2-methyl-2-oxazoline)-	poly(2-methyl-2-oxazoline)-*b*-poly(γ-benzyl-l-glutamate), poly(2-methyl-2-oxazoline)-*b*-poly(phenylalanine), poly(2-methyl- 2-oxazoline)-*b*-poly[*O*-(tetra-*O*-acetyl--d-glucopyranosyl)-l-serine], and poly(2-phenyl-2-oxazoline)-*b*-poly[*O*-(tetra*O*-acetyl--d-glucopyranosyl)-l-serine]	[170,171]
Diblock	Poly(methyl acrylate)	poly(methyl acrylate)-*b*-poly(γ-benzyl-l-glutamate)	[146]
Diblock	Polystyrene	polystyrene-*b*-Poly(Z-l-Lysine)	[68,160]
Triblock	Poly(l-2-anthraquinonylalanine)	poly(γ-benzyl-l-glutamate)-*b*-poly(l-2-anthraquinonylalanine)-*b*-poly(γ-benzyl-l-glutamate)	[172]
Triblock copolymer	Polyethylene oxide	poly(γ-benzyl-l-glutamate)-*b*-polyethyelene oxide-*b*-poly(γ-benzyl-l-glutamate	[173]
Triblock copolymer	Polyethylene oxide	poly(Z-l-Lysine)-*b*-polyethyelene oxide-*b*-Poly(Z-l-Lysine)	[174,175]
Triblock copolymer	Polyethylene oxide	poly[(l-valine)-*co*-(l-leucine)]-*b*-polyethyelene oxide-*b*-poly[(l-valine)-*co*-(l-leucine)]	[166]
Triblock copolymer	Polyethylene oxide/Polylactic acid	polyethylene oxide-*b*-Polylactic acid-*b*-poly(γ-benzyl-l-glutamate)	[176]
Triblock terpolymers	Polyethylene oxide	polyethyelene oxide-*b*-Poly(Z-l-Lysine)-*b*-poly(γ-benzyl-l-glutamate)	[153]
Pentablock	Polystyrene	poly(ε-tert-butyloxycarbonyl-l-lysine)]-*b*-poly(γ-benzyl-l-glutamate)-*b*-Polystyrene-*b* poly(γ-benzyl-l-glutamate)-*b*-poly(ε-tert-butyloxycarbonyl-l-lysine)]	[177]

**Table 5:** Summary of strategies and aminoacids employed to fabricate cyclic polypeptides.

Amino Acid *N*-Carboxyanhydride(s) Employed	Conditions	References
d,l-Leucine-NCA, and d,l-Phenylalanine-NCA	Dioxane at 60 °C/Initiator: imidazole	[179]
d,l-alanine *N*-carboxyanhydride, d,l-phenylalanine *N*-carboxyanhydride, and d,l-leucine *N*-carboxyanhydride	Using pyridine as initiator.	[178]
Sarcosine-NCA at 120 °C	Thermal polymerization	[180]
d,l-Phenylalanine-NCA	Difunctional primary amines (1,12-diaminododecane, DAD, or 1,13-diamino-4,7,10-trioxatridecane, DATT) for the synthesis of linear well-defined telechelic polymers bearing amino end groups. Subsequent reaction of the telechelic polymers with 4,4′-Diisocyanatodiphenylmethane lead to cyclic polypeptides	[181]
